# Inhibition of heat shock proteins increases autophagosome formation, and reduces the expression of APP, Tau, SOD1 G93A and TDP-43

**DOI:** 10.18632/aging.203297

**Published:** 2021-07-12

**Authors:** Paul Dent, Laurence Booth, Jane L. Roberts, Andrew Poklepovic, Derek Cridebring, Eric M. Reiman

**Affiliations:** 1 Department of Biochemistry and Molecular Biology, Richmond, VA 23298, USA; 2 Department of Pharmacology and Toxicology, Richmond, VA 23298, USA; 3 Department of Medicine, Virginia Commonwealth University, Richmond, VA 23298, USA; 4 Translational Genomics Research Institute (TGEN), Phoenix, AZ 85004, USA; 5 Banner Alzheimer’s Institute, Phoenix, AZ 85006, USA

**Keywords:** Alzheimer's, chaperone, GRP78, autophagy, neratinib

## Abstract

Aberrant expression and denaturation of Tau, amyloid-beta and TDP-43 can lead to cell death and is a major component of pathologies such as Alzheimer’s Disease (AD). AD neurons exhibit a reduced ability to form autophagosomes and degrade proteins via autophagy. Using genetically manipulated colon cancer cells we determined whether drugs that directly inhibit the chaperone ATPase activity or cause chaperone degradation and endoplasmic reticulum stress signaling leading to macroautophagy could reduce the levels of these proteins. The antiviral chaperone ATPase inhibitor AR12 reduced the ATPase activities and total expression of GRP78, HSP90, and HSP70, and of Tau, Tau 301L, APP, APP692, APP715, SOD1 G93A and TDP-43. In parallel, it increased the phosphorylation of ATG13 S318 and eIF2A S51 and caused eIF2A-dependent autophagosome formation and autophagic flux. Knock down of Beclin1 or ATG5 prevented chaperone, APP and Tau degradation. Neratinib, used to treat HER2+ breast cancer, reduced chaperone levels and expression of Tau and APP via macroautophagy, and neratinib interacted with AR12 to cause further reductions in protein levels. The autophagy-regulatory protein ATG16L1 is expressed as two isoforms, T300 or A300: Africans trend to express T300 and Europeans A300. We observed higher basal expression of Tau in T300 cells when compared to isogenic A300 cells. ATG16L1 isoform expression did not alter basal levels of HSP90, HSP70 or HSP27, however, basal levels of GRP78 were reduced in A300 cells. The abilities of both AR12 and neratinib to stimulate ATG13 S318 and eIF2A S51 phosphorylation and autophagic flux was also reduced in A300 cells. Our data support further evaluation of AR12 and neratinib in neuronal cells as repurposed treatments for AD.

## INTRODUCTION

The pathologies Alzheimer’s Disease (AD), familial Amyotrophic Lateral Sclerosis (ALS) and Huntington’s Chorea (HC) are all in part mediated by the aberrant expression and denaturation of disease-specific proteins [1–6]. In AD, amyloid-β and Tau proteins form toxic aggregates inside neurons and in the extracellular environment. The majority of familial ALS is caused by expression of mutant variants of SOD1, e.g., SOD1 G93A. Toxic protein aggregates, such as those containing TDP-43, FUS and SOD1 G93A, are causal in neuronal cell death. An additional major component of brain biology in ALS patients are activated / reactive microglia [7, 8]. The reactive microglia create a toxic inflammatory micro-environment liminal to the dysfunctional neurons. In HC, multiple additional genetically unstable CAG repeats result in translation of a Huntingtin protein which is more readily cleaved by cellular proteases to form insoluble toxic aggregates in neurons.

Protein expression and protein degradation are tightly controlled processes. An important regulator of protein translation is the factor eIF2α [9–12]. Phosphorylation of eIF2α S51 inactivates its function, and bulk protein translation is reduced. Several kinases phosphorylate eIF2α, including PKR-like endoplasmic reticulum kinase (PERK). The bi-functional protein chaperone GRP78 binds to PERK, keeping it inactive [13–16]. GRP78 senses the amount of denatured protein in the ER and cytosol, and as the levels of denatured proteins increase, GRP78 is released from PERK, and then acts as a chaperone to renature the misfolded proteins. PERK becomes active, phosphorylates eIF2α thus stopping translation until GRP78 and other chaperone proteins of the HSP90 and HSP70 families have renatured the misfolded proteins. GRP78 then can re-associate with PERK, inactivating the kinase, and eIF2α is dephosphorylated and activated by protein serine/threonine phosphatase 1.

Autophagy is an evolutionary conserved mechanism by which cells recycle nutrients to maintain viability [17–22]. Proteins that cannot be renatured by chaperones are degraded via autophagy (along with the associated chaperone protein). Although phosphorylation of eIF2α inhibits translation from ~95% of genes, it also stimulates translation of alternate genes including the autophagosome regulatory proteins Beclin1 and ATG5 [23]. Hence ER stress signaling to restore homeostasis acts to both renature proteins and simultaneously to facilitate the break down excess protein load via autophagy. In AD the clearance of amyloid-β plaques can be mediated via autophagy, which correlates with improved *in vivo* mouse memory function [22, 24]. Similar *in vitro* and *in vivo* findings to amyloid-β have been made for ALS aggregates and mutant Huntingtin aggregates [21, 25].

Our prior studies in oncology, specifically developmental cancer therapeutics, have shown how drugs such as neratinib and niraparib, which induce ER stress and cause autophagosome formation through different mechanisms, can be combined to synergistically kill tumor cells, e.g. [26, 27]. Several of these concepts have been translated into the clinic [28, 29]. The drug AR12 was originally developed and translated into the clinic as a cancer therapeutic which killed cells via autophagy and ER stress signaling, however, when its true mechanism of action was discovered, as a pan-chaperone ATPase inhibitor, and particularly as an inhibitor of GRP78, the drug was repurposed into the infectious disease field as an anti-viral drug [NCT00978523, ASCO Abs. #2068, 2014; 30–37]. AR12 prevents the replication of viruses such as SARS-CoV-2 by preventing them from hijacking chaperone functions [38–40]. These attributes suggest to us that AR12 may have utility as an AD therapeutic by causing ER stress and autophagy. In the present studies we have used isogenic HCT116 colon cancer cells as a model system to examine these processes.

Recent studies have shown that HER2 and HER4 are over-expressed in the hippocampus of human AD brains and that degradation of HER2 enhances autophagic flux, causes degradation of amyloid-β, and improves the cognitive functions of APP/presenilin-1 (PS1) mice [41, 42]. Neratinib down-regulates RAS expression and signaling by RAS proteins has been linked to increased expression of Tau and amyloid-β proteins [43, 44]. Neratinib also inactivates the Hippo pathway and recently Hippo signaling was shown to be activated in amyloid-β-mediated neurodegeneration and that inhibition of Hippo signaling rescues the neurodegeneration effect [45, 46]. As neratinib down-regulates HER2, HER4 and RAS, inactivates Hippo and inactivates mTOR and causes autophagosome formation, we also hypothesized repurposed neratinib could safely reduce misfolded protein levels in AD.

Our oncology studies revealed that the multiple sclerosis drugs fingolimod and dimethyl fumarate interacted to kill glioblastoma cells which was mediated by increased autophagosome formation [47, 48]. The drugs prevented freshly isolated reactive microglia, from a patient GBM tumor, from synthesizing IL-6, TNFα and TGFβ. As the pathologies of AD, ALS and HC all involve sustained expression of denatured toxic proteins, and as our oncology and virology-based drug combinations stimulate autophagosome formation, ER stress and reduced protein translation and autophagic digestion of proteins, we determined whether our repurposed drug combinations using transformed / tumor cell systems, not neurons, could reduce expression of Tau and APP via autophagy.

## MATERIALS AND METHODS

### Materials

**Figure 1:**
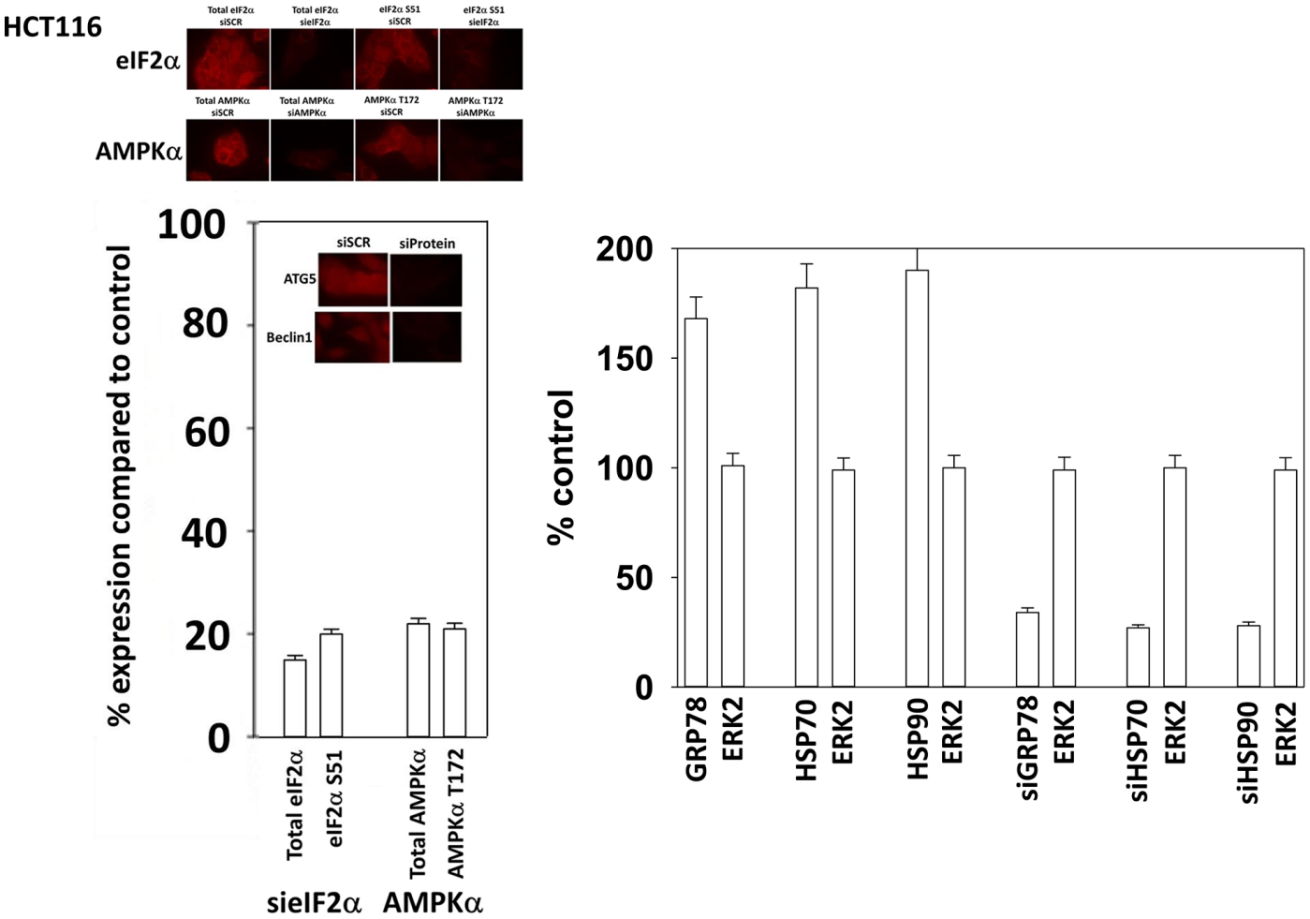
**Control studies showing siRNA knockdown of proteins in HCT116 cells.** HCT116 cells were transfected with a scrambled control siRNA or siRNA molecules to knock down the expression of eIF2α, AKPKα_1_, Beclin1 or ATG5. Twenty-four h afterwards, cells were fixed in place and the expression and phosphorylation of the indicated proteins determined.

HCT116 ATG16L1 T300 cells were purchased by Dr. David L. Boone (University of Notre Dame, South Bend, OH) and were used to generate isogenic HCT116 ATG16L1 A300 knock in cells [49]. African Green monkey kidney (Vero) cells were a kind gift of Dr. Jin Kim (University of South Alabama, Mobile, AL). GBM6 cells were kindly provided by the Mayo Clinic cell bank (Rochester, MN). AR12 and MMF were purchased from Selleckchem (Houston, TX). Neratinib was supplied by Puma Biotechnology Inc. (Los Angeles, CA). Fingolimod (FTY720) was purchased from Sigma-Aldrich (St. Louis MO). Plasmids to express LC3-GFP-RFP, Tau-GFP, Tau 301L-GFP, APP-FLAG, APP715-FLAG, APP692-FLAG, SOD1 G93A, TDP-43 and Huntingtin CAG145 were purchased from Addgene (Watertown, MA). The plasmid to express the SARS-CoV-2 spike protein was from Sino Biological (Wayne, PA). Trypsin-EDTA, RPMI, penicillin-streptomycin were purchased from GIBCOBRL (GIBCOBRL Life Technologies, Grand Island, NY). Other reagents and performance of experimental procedures were as described [26, 31, 36, 37, 43, 46, 48, 50–52]. Antibodies used: Beclin1 (3495), eIF2α (5324), AMPKα (2532), ATG5 (12994), ATG13 (13468) all from Cell Signaling Technology (Danvers, MA); P-ATG13 S318 (19127) from Novus Biologicals. Specific multiple independent siRNAs to knock down the expression of AMPKα_1_, Beclin1, ATG5 and eIFα, and scramble control, were purchased from Qiagen (Hilden, Germany). Additional antibodies used in this study: HSP90 (E289) (Cell Signaling); HSP90 (#2928) (Abcam); HSP90 (ab195575) Abcam; HSP90 3G3 (13495) (Abcam); GRP78 (50b12) (31772) (Cell Signaling); GRP78 (ab191023) Abcam; GRP78 (ab103336) Abcam; GRP78 (N-20) (sc-1050) Santa Cruz; HSP27 (G31) (2402P) Cell Signaling); HSP27 [EP1724Y] (ab62339) Abcam; HSP27 (H-77) (sc-9012) Santa Cruz; HSP27 (LS-C31836). Multiple control studies have been previously presented showing on-target specificity of our siRNAs, primary antibodies, and our phospho-specific antibodies to detect both total protein levels and phosphorylated levels of proteins (Figure 1) [51, 52].

### Methods

All bench-side Methods used in this manuscript have been performed and described in the peer-reviewed references [26, 31, 36, 37, 43, 46, 48, 50–52]. All cell lines were cultured at 37° C (5% (v/v CO_2_) *in vitro* using RPMI supplemented with dialyzed 5% (v/v) fetal calf serum and 1% (v/v) Non-essential amino acids. Drugs are dissolved in DMSO to make 10 mM stock solutions. The stock solution is diluted to the desired concentration in the media that the cells being investigated grow in. We ensure that the concentration of DMSO is never more than 0.1% (v/v) in the final dilution that is added to cells, to avoid solvent effects. Cells were never cultured in reduced serum media.

### Assessments of protein expression and protein phosphorylation

[26, 31, 36, 37, 43, 46, 48, 50–52]. Multi-channel fluorescence HCS microscopes perform true in-cell western blotting. Three independent cultures derived from three thawed vials of cells of a tumor were sub-cultured into individual 96-well plates. Twenty-four hours after plating, the cells are transfected with a control plasmid or a control siRNA, or with an empty vector plasmid or with plasmids to express various proteins. After another 24 hours, the cells are ready for drug exposure(s). At various time-points after the initiation of drug exposure, cells are fixed in place using paraformaldehyde and using Triton X100 for permeabilization. Standard immunofluorescent blocking procedures are employed, followed by incubation of different wells with a variety of validated primary antibodies and subsequently validated fluorescent-tagged secondary antibodies are added to each well. The microscope determines the background fluorescence in the well and in parallel randomly determines the mean fluorescent intensity of 100 cells per well. Of note for scientific rigor is that the operator does not personally manipulate the microscope to examine specific cells; the entire fluorescent accrual method is independent of the operator.

### Transfection of cells with siRNA or with plasmids

### For plasmids

Cells were plated and 24h after plating, transfected. Plasmids expressing a specific mRNA or appropriate empty vector control plasmid (CMV) DNA was diluted in 50 μl serum-free and antibiotic-free medium (1 portion for each sample). Concurrently, 2 μl Lipofectamine 2000 (Invitrogen), was diluted into 50 μl of serum-free and antibiotic-free medium (1 portion for each sample). Diluted DNA was added to the diluted Lipofectamine 2000 for each sample and incubated at room temperature for 30 min. This mixture was added to each well / dish of cells containing 100 μl serum-free and antibiotic-free medium for a total volume of 300 μl, and the cells were incubated for 4 h at 37° C. An equal volume of 2x serum containing medium was then added to each well. Cells were incubated for 24h, then treated with drugs.

### Transfection for siRNA

Cells from a fresh culture growing in log phase as described above, and 24h after plating transfected. Prior to transfection, the medium was aspirated, and serum-free medium was added to each plate. For transfection, 10 nM of the annealed siRNA or the negative control (a “scrambled” sequence with no significant homology to any known gene sequences from mouse, rat or human cell lines) were used. Ten nM siRNA (scrambled or experimental) was diluted in serum-free media. Four μl Hiperfect (Qiagen) was added to this mixture and the solution was mixed by pipetting up and down several times. This solution was incubated at room temp for 10 min, then added dropwise to each dish. The medium in each dish was swirled gently to mix, then incubated at 37° C for 2h. Serum-containing medium was added to each plate, and cells were incubated at 37° C for 24h before then treated with drugs (0-24h).

### Assessments of autophagosome and autolysosome levels

Cells were transfected with a plasmid to express LC3-GFP-RFP, and as indicated with siRNA molecules. Twenty-four h after transfection, cells are treated with vehicle control or the drugs, alone or in combination as indicated. Cells were imaged at 60X magnification 4 h and 8 h after drug exposure and the mean number of GFP+ and RFP+ punctae per cell determined from >50 randomly selected cells per condition. With three independent triplicates used to calculate the mean number of punctae per cell.

### Assessments of protein expression and protein phosphorylation

[26, 31, 36, 37, 43, 46, 48, 50–52]. Multi-channel fluorescence HCS microscopes perform true in-cell western blotting. Three independent cultures derived from three thawed vials of cells of a tumor were sub-cultured into individual 96-well plates. Twenty-four hours after plating, the cells are transfected with a control plasmid or a control siRNA, or with an empty vector plasmid or with plasmids to express various proteins. After another 24 hours, the cells are ready for drug exposure(s). At various time-points after the initiation of drug exposure, cells are fixed in place using paraformaldehyde and using Triton X100 for permeabilization. Standard immunofluorescent blocking procedures are employed, followed by incubation of different wells with a variety of validated primary antibodies and subsequently validated fluorescent-tagged secondary antibodies are added to each well. The microscope determines the background fluorescence in the well and in parallel randomly determines the mean fluorescent intensity of 100 cells per well. Of note for scientific rigor is that the operator does not personally manipulate the microscope to examine specific cells; the entire fluorescent accrual method is independent of the operator.

### Data analysis

Comparison of the effects of various treatments was using one-way ANOVA for normalcy followed by a two tailed Student’s t-test with multiple comparisons. Differences with a p-value of < 0.05 were considered statistically significant. Experiments are the means of multiple individual data points per experiment from 3 independent experiments (± SD).

## RESULTS

**Figure 2:**
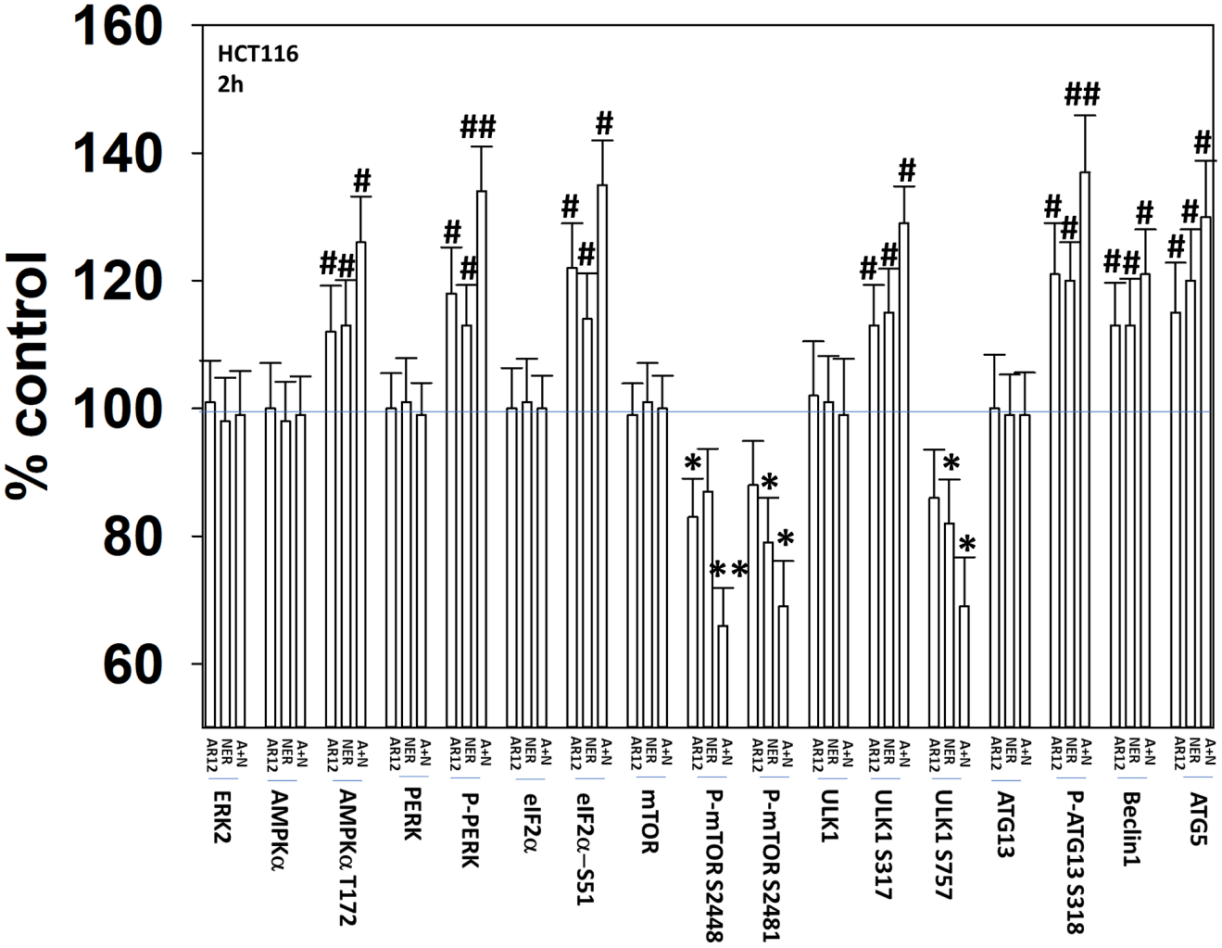
**AR12 and neratinib interact to cause greater activation of PERK and ATG13 and inactivation of mTORC1.** HCT116 ATG16L1 T300 cells were treated with vehicle control, neratinib (50 nM), AR12 (2.0 μM) or the drugs in combination for 2h. Cells were fixed in place and immunostaining performed. Data are presented as the percentage staining intensity compared to vehicle control (defined as 100%) (n = 3 +/-SD) * p < 0.05 less than vehicle control value; # p < 0.05 greater than vehicle control value; ** p < 0.05 less than either individual treatment value; ## p < 0.05 greater than either individual treatment value.

Our initial studies were designed to demonstrate that AR12 and neratinib, and fingolimod and MMF, interacted to cause autophagosome formation and autophagic flux. Treatment of HCT116 ATG16L1 T300 colon cancer cells with AR12 or neratinib increased the activities of the AMPK, ULK1 and ATG13 (Figure 2). The activities of mTORC1 and mTORC2 declined. The drugs combined to cause further activation of PKR-like endoplasmic reticulum kinase (PERK) that was associated with increased phosphorylation, and inactivation of eIF2α.

**Figure 3:**
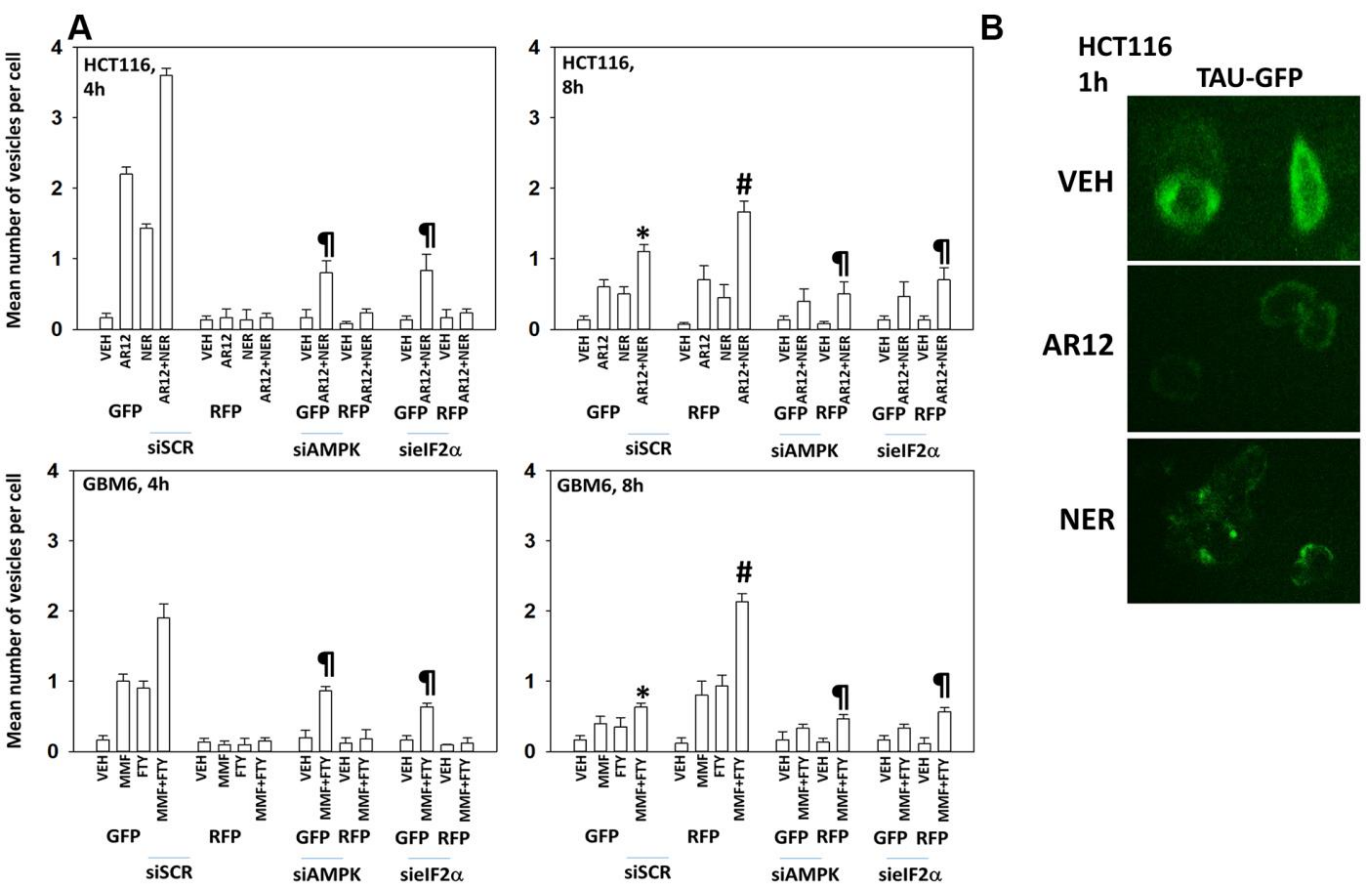
**Drug interactions promoting eIF2α-dependent autophagosome formation and autophagic flux.** (**A**) HCT116 ATG16L1 T300 and GBM6 cells were transfected with a plasmid to express LC3-GFP-RFP and in parallel with a scrambled siRNA or with siRNA molecules to knock down the expression of eIF2α or AMPKα_1_. After 24h, cells were treated with vehicle control, AR12 (2 μM), neratinib (50 nM), MMF (5 μM), fingolimod (FTY, 100 nM) or the drugs in combination as indicated in the graphs. Randomly cells (> 50 per data point) were examined 4h and 8h after drug exposure and the mean number of GFP+ and RFP+ intense staining punctae determined under each condition (n = 3 +/-SD) * p < 0.05 less than corresponding value after 4h; # p < 0.05 greater than corresponding value after 4h; ¶ p < 0.05 less than corresponding value in siSCR cells. (**B**) HCT116 ATG16L1 T300 cells were transfected with a plasmid to express wild type Tau-GFP. After 24h, cells were treated with vehicle control, AR12 (2 μM) or neratinib (50 nM). Cells were fixed after 1h. Cells were fixed in place and the detection of Tau levels determined using GFP tag fluorescence (10X).

HCT116 ATG16L1 T300 colon cancer cells and GBM6 cells, a PDX glioblastoma isolate expressing truncated EGFR vIII, were transfected with a plasmid to express LC3-GFP-RFP and with siRNA molecules to knock down expression of AMPKα_1_ or eIF2α. In the neutral autophagosome both GFP and RFP fluoresce whereas in the acidic autolysosome GFP is quenched and only RFP fluoresces. Thus, the appearance and disappearance of GFP+/RFP+ and RFP+ vesicles over time permits the detection of autophagosome formation and autophagic flux where autophagosomes fuse with acidic lysosomes to form autolysosomes. AR12 and neratinib, in agreement with the elevated phosphorylation of ATG13, both increased autophagosome formation in the HCT116 cells within 4h of exposure and combined to further promote autophagosome formation (Figure 3A, upper graphs). Autophagosome formation was significantly reduced by knock down of AMPKα_1_ or eIF2α. Eight hours after drug exposure, the levels of autophagosomes had declined and the numbers of RFP+ autolysosomes had increased, with this effect also being significantly suppressed by knock down of AMPKα_1_ or eIF2α.

In transfected GBM6 cells, fingolimod and MMF both enhanced autophagosome formation, with the combination causing greater levels of autophagy, and these effects were blocked by knock down of AMPKα_1_ or eIF2α (Figure 3A, lower graphs). As was observed in the upper graphs, the fingolimod / MMF drug combination caused autophagic flux which was again blocked by knock down of AMPKα_1_ or eIF2α. From Addgene we purchased plasmids to express a Tau-GFP fusion protein (“wild type”) and to express a mutant Tau 301L-GFP fusion protein (“mutant”). HCT116 cells were transfected with a plasmid to express “wild type” Tau-GFP and treated with AR12 or neratinib. Treatment with AR12 reduced Tau-GFP levels, as did neratinib (Figure 3B). AR12 did not apparently alter Tau sub-cellular localization. Our prior studies with neratinib had demonstrated that the drug rapidly caused HER family receptors and RAS proteins to become localized in punctate intracellular vesicles, and in the present studies, neratinib, unlike AR12, also caused Tau-GFP to rapidly exhibit a punctate staining pattern [43].

**Figure 4:**
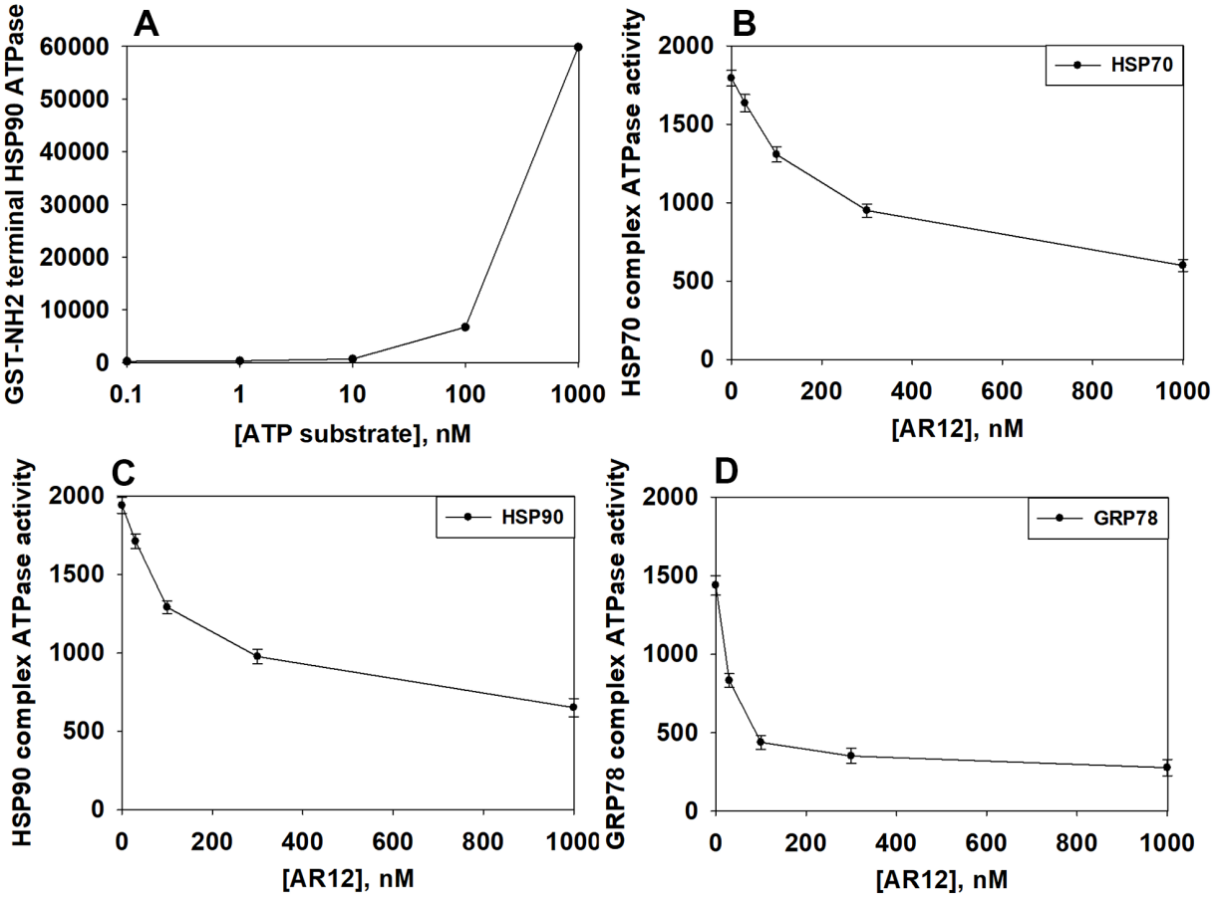
**AR12 inhibits the chaperone ATPase activities of HSP90, HSP70 and GRP78.** (**A**) A GST-HSP90 NH2-terminal fragment containing the ATP binding domain of the chaperone was synthesized in E. coli and purified from other bacterial proteins using glutathione Sepharose. The GST-HSP90 NH2-terminal fragment protein was not eluted off the Sepharose beads. Equal portions of beads were immediately aliquoted into individual wells in a 96 well plate. Beads were resuspended in kinase reaction buffer in triplicate, and incubated for 30 min at 37° C. The reaction was started by addition of increasing concentrations of the ATP-lite substrate. The plate was removed from the incubator and placed into a Vector 3 plate reader to determine the luminescence of the reactions under each condition (n = 3 (× 3) +/-SD). (**B**, **C**) GBM12 cells were transfected with a plasmid to express HSP70-GFP or to express FLAG-tagged HSP90. Twenty-four h after transfection chaperone proteins were immuno-precipitated using their tags in the presence of phosphatase inhibitors. Equal portions of precipitate Sepharose beads were immediately aliquoted into individual wells in a 96 well plate. Beads were resuspended in ATPase reaction buffer containing vehicle control or AR12 (30 nM; 100 nM; 300 nM; 1 μM) in triplicate, and incubated for 30 min at 37° C. The reaction was started by addition of ATP-lite substrate (2 μM). The plate was removed from the incubator and placed into a Vector 3 plate reader to determine the luminescence of the reactions under each treatment condition (n = 3 (× 3) +/-SD). (**D**) GBM12 cells were transfected with a plasmid to express GRP78. Twenty-four h after transfection GRP78 was immuno-precipitated using an antibody directed to the COOH terminal portion of the protein in the presence of phosphatase inhibitors. Equal portions of precipitate Sepharose beads were immediately aliquoted into individual wells in a 96 well plate. Beads were resuspended in ATPase reaction buffer containing vehicle control or AR12 (30 nM; 100 nM; 300 nM; 1 μM) in triplicate, and incubated for 60 min at 37° C. The reaction was started by addition of ATP-lite substrate (2 μM). The plate was removed from the incubator and placed into a Vector 3 plate reader to determine the luminescence of the reactions under each treatment condition (n = 3 (× 3) +/-SD).

We then performed studies to define dose responses for AR12 inhibiting the chaperone ATPase activities of HSP90, HSP70 and GRP78. As an internal control, we observed a concentration-dependent increase in fluorescence from the ATP-lite substrate (Figure 4A). In a dose-dependent fashion AR12 inhibited the ATPase activities of HSP90, HSP70 and GRP78 (Figure 4B–4D). Of note was that the inherent ATPase activity of GRP78 in our analyses was considerably lower than those of HSP70 and HSP90; the basal activity was significantly less as measured by the assay and was generated by twice as long an incubation compared to HSP90 and HSP70. The approximate IC50 inhibitory concentration for HSP70 was ~400 nM, for HSP90 ~300 nM and for GRP78 ~80 nM. In heavily pre-treated cancer patients treated with AR12, the C max of the drug at the RP2D of 800 mg BID varied between 2 μM and 8 μM. We previously published that AR12 crossed the blood-brain barrier, with a brain C max of ~2 μM after administration of a 50 mg/kg bolus [37]. Collectively, our findings support the use of AR12 as a therapeutic anti-chaperone agent in the brain.

**Figure 5:**
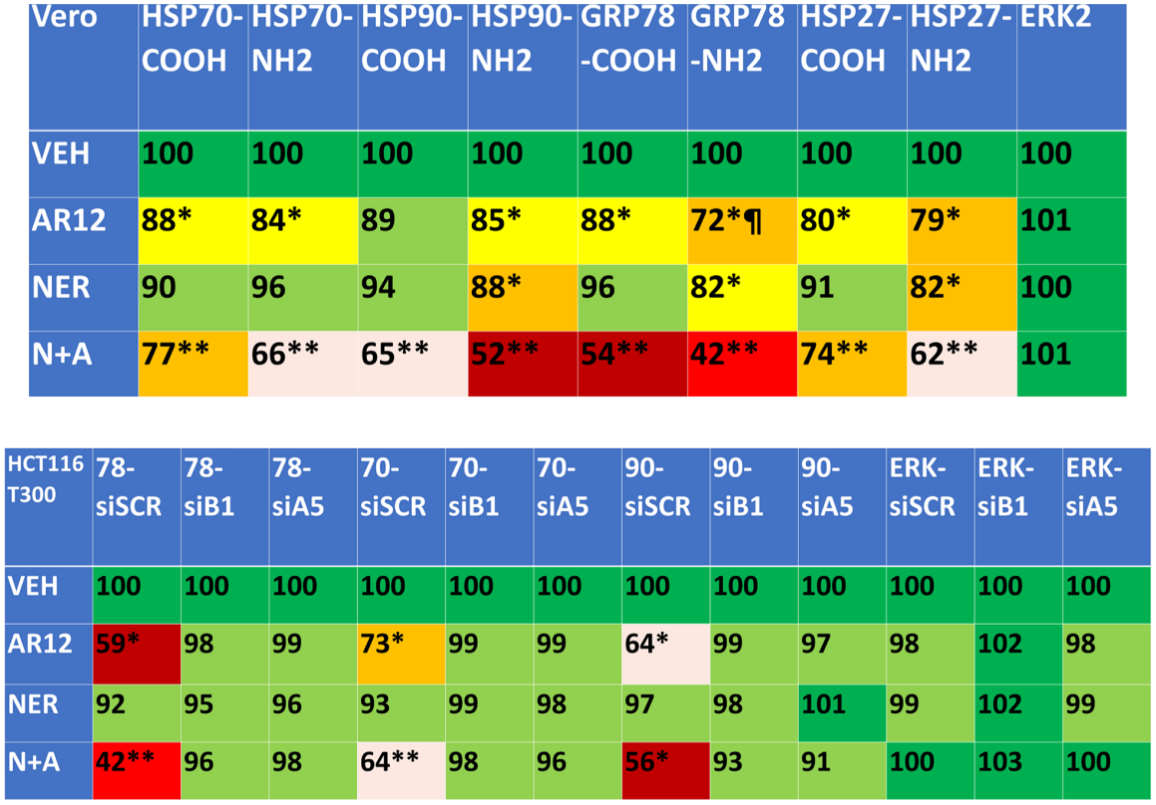
**Neratinib and AR12 combine to reduce the expression of HSP90, HSP70, GRP78 and HSP27 via autophagy.** Upper: Vero cells were treated with vehicle control, AR12 (2 μM), neratinib (50 nM) or the drugs in combination for 6h. Cells were fixed in place and in cell immunostaining performed to determine chaperone expression using antibodies whose epitopes are localized at the NH2- and COOH-termini of each protein (n = 3 +/-SD) * p < 0.05 less than vehicle control; ** p < 0.05 less than neratinib single agent value; ¶ p < 0.05 less than value determined using the COOH-terminal epitope antibody. Lower: HCT116 ATG16L1 T300 cells were transfected with a scrambled siRNA or with siRNA molecules to knock down the expression of Beclin1 or ATG5. After 24h, cells were treated with vehicle control, AR12 (2 μM), neratinib (50 nM) or the drugs in combination for 6h. Cells were fixed in place and in cell immunostaining performed to determine chaperone expression using antibodies whose epitopes is at the COOH-termini of each protein (n = 3 +/-SD) * p < 0.05 less than vehicle control; ** p < 0.05 less than neratinib single agent value.

We next defined the impact of neratinib and AR12 as single agents and when combined on the expression of protein chaperones. AR12 significantly reduced chaperone expression whether the levels were measured using an antibody directed at the COOH terminus or NH2 terminus of the protein (Figure 5, upper). Notably for GRP78 the reduced staining for the NH2 terminus of the protein did not match that observed for the COOH terminal epitope, recapitulating our prior studies that AR12 binds to the ATP binding domain of GRP78 and alters protein conformation such that the NH2 antibody epitope has become occluded. For all of the tested chaperones, including HSP27 that does not bind ATP, neratinib and AR12 interacted to cause a significant further reduction in chaperone levels. We then determined whether autophagosome formation was responsible for the drug-induced decline in chaperone expression. Knock down of Beclin1 or ATG5 prevented neratinib and AR12 from reducing chaperone expression (Figure 5, lower). Thus, not only does AR12 catalytically inhibit chaperones, but the drug also causes their degradation through autophagy.

**Figure 6:**
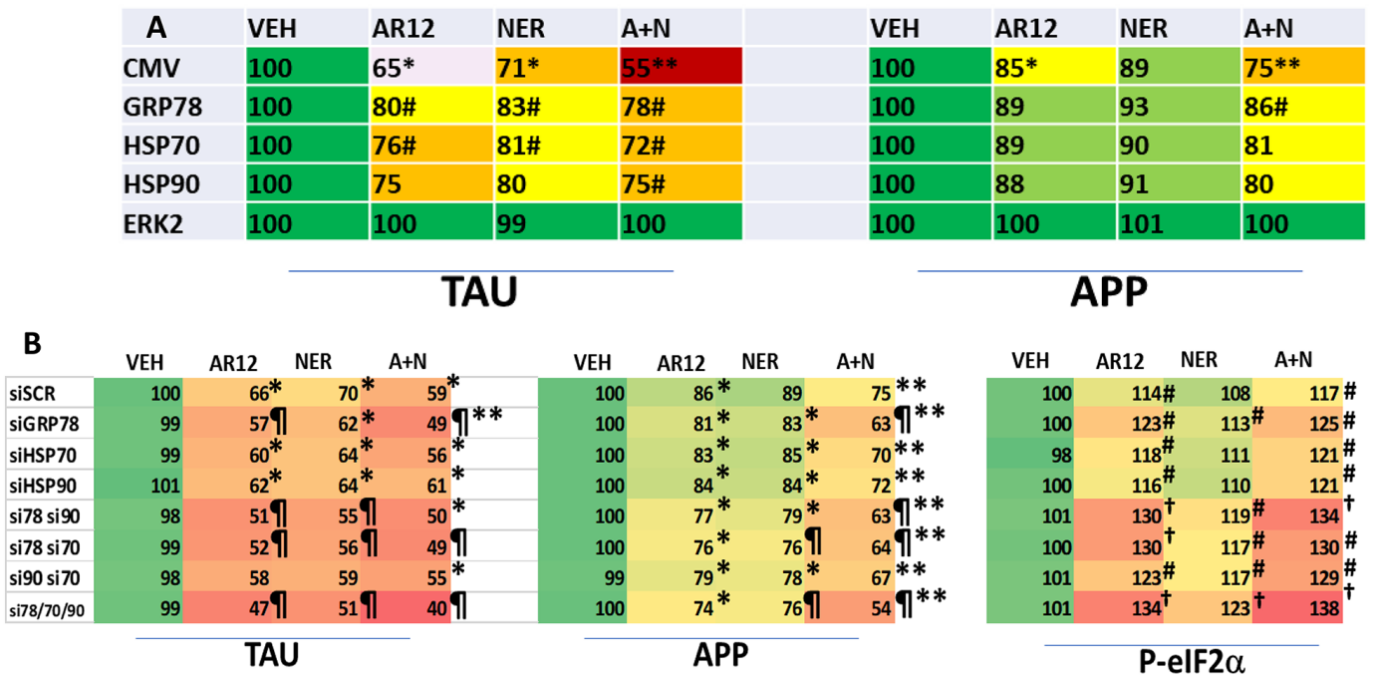
**GRP78, HSP90 and HSP70 play important roles in maintaining the stability of Tau and APP.** (**A**) HCT116 ATG16L1 T300 cells were transfected with an empty vector plasmid (CMV) or with plasmids to express GRP78, HSP70 or HSP90, and were co-transfected to express either Tau or APP. Twenty-four h after transfection cells were treated with vehicle control, AR12 (2 μM), neratinib (50 nM) or the drugs in combination for 24h. Cells were fixed in place and the expression of Tau and APP determined (n = 3 +/-SD). # p < 0.05 greater than corresponding value in CMV transfected cells; * p < 0.05 less than corresponding value in corresponding vehicle control cells; ** p < 0.05 less than corresponding AR12 value. (**B**) HCT116 ATG16L1 T300 cells were transfected with a scrambled control siRNA (siSCR) or with siRNA molecules to knock down expression of GRP78, HSP70 or HSP90, as indicated in the panel. In parallel, cells were transfected to express either Tau or APP. Twenty-four h after transfection, cells were fixed in place and the expression of Tau and APP determined (n = 3 +/-SD). Vehicle control transfected with a scrambled siRNA is defined at 100%. * p < 0.05 less than corresponding value in vehicle control cells; ** p < 0.05 less than corresponding AR12 value; p < 0.05 less than corresponding value in siSCR cells; † p < 0.05 greater than corresponding value in siSCR cells.

We next performed studies to link chaperone proteins to the expression and drug-induced degradation of Tau and APP. Cells were transfected to over-express GRP78, HSP90 or HSP70 and co-transfected to express Tau or APP. Over-expression of GRP78 and to a lesser extent HSP70 both significantly reduced the ability of AR12, neratinib and the drugs in combination to lower the levels of Tau (Figure 6A). Only GRP78 over-expression significantly reduced the abilities of the drugs as single agents or combined to lower APP expression. Cells were then transfected with siRNA molecules to knock down the expression of GRP78, HSP90 or HSP70, and in combination, and co-transfected to express Tau or APP, followed by drug exposure. Surprisingly, under basal conditions, knock down of GRP78 / HSP90 / HSP70 did not significantly alter the basal levels of Tau or APP expression or the basal levels of sIF2α S51 phosphorylation (Figure 6B). Knock down of GRP78 significantly enhanced the ability of AR12 and [AR12 + neratinib] to reduce the expression of Tau and APP. Knock down of GRP78 in combination with knock down of HSP90 or HSP70 caused significantly more protein degradation than GRP78 alone and a significantly greater amount of eIF2α phosphorylation.

**Figure 7:**
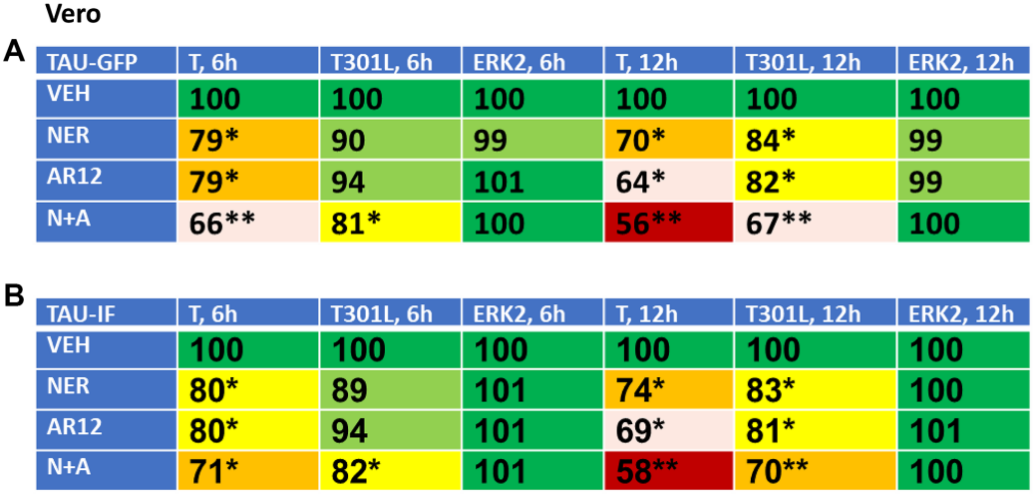
**Neratinib and AR12 interact to reduce the expression of Tau and Tau 301L in Vero cells.** (**A**, **B**) Vero cells were transfected with plasmids to express Tau-GFP or Tau 301L-GFP. After 24h, cells were treated for 6h or 12h with vehicle control, AR12 (2 μM), neratinib (50 nM) or the drugs in combination. Cells were fixed in place and the detection of Tau levels determined using either GFP tag fluorescence or by in cell immunostaining (n = 3 +/-SD) * p < 0.05 less than vehicle control; ** p < 0.05 less than neratinib single agent value.

**Figure 8:**
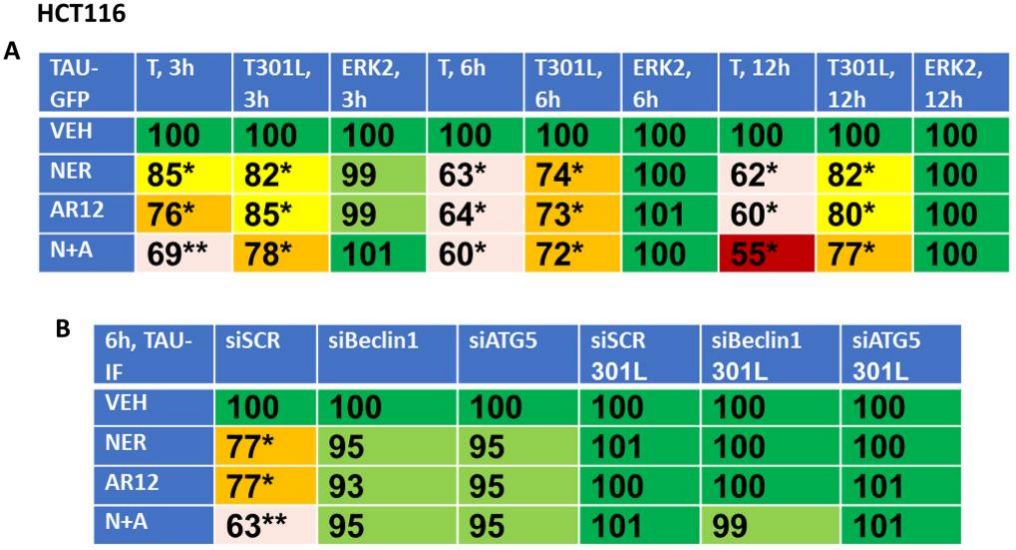
**Neratinib and AR12 interact to reduce the expression of Tau and Tau 301L via autophagy.** (**A**) HCT116 cells were transfected with plasmids to express Tau-GFP or Tau 301L-GFP. After 24h, cells were treated for 3h, 6h or 12h with vehicle control, AR12 (2 μM), neratinib (50 nM) or the drugs in combination. Cells were fixed in place and the detection of Tau levels determined using GFP tag fluorescence (n = 3 +/-SD) * p < 0.05 less than vehicle control; ** p < 0.05 less than neratinib single agent value. (**B**) HCT116 ATG16L1 T300 cells were transfected to express Tau-GFP or Tau 301L-GFP and in parallel with a scrambled siRNA or with siRNA molecules to knock down the expression of Beclin1 or ATG5. After 24h, cells were treated with vehicle control, AR12 (2 μM), neratinib (50 nM) or the drugs in combination for 6h. Cells were fixed in place and in cell immunostaining performed to determine Tau expression (n = 3 +/-SD) * p < 0.05 less than vehicle control; ** p < 0.05 less than neratinib single agent value.

Based on the data in Figure 6, we reasoned that if neratinib and AR12 could inhibit chaperone function and rapidly stimulate autophagosome formation, the drug combination would also be able to degrade other proteins including “wild type” and “mutant” Tau and APP proteins. African Green monkey kidney (Vero) cells and human HCT116 ATG16L1 T300 colon cancer cells were transfected to express “wild type” Tau or a “mutant” Tau, Tau 301L, which has been stated to be relatively protease resistant compared to the wild type protein, with both proteins tagged with GFP [53, 54] (Figures 7, 8). Over a time-course, neratinib and AR12 interacted to reduce the expression of wild type Tau or Tau 301L, either via GFP tag fluorescence or by immunostaining (Figures 7, 8). Transfection of the human HCT116 cells to knock down Beclin1 or ATG5 prevented the drug combination from reducing Tau or Tau 301L expression. Hence, we degrade not only the chaperones which associate with Tau proteins, but also the Tau protein itself (Figure 8B).

**Figure 9:**
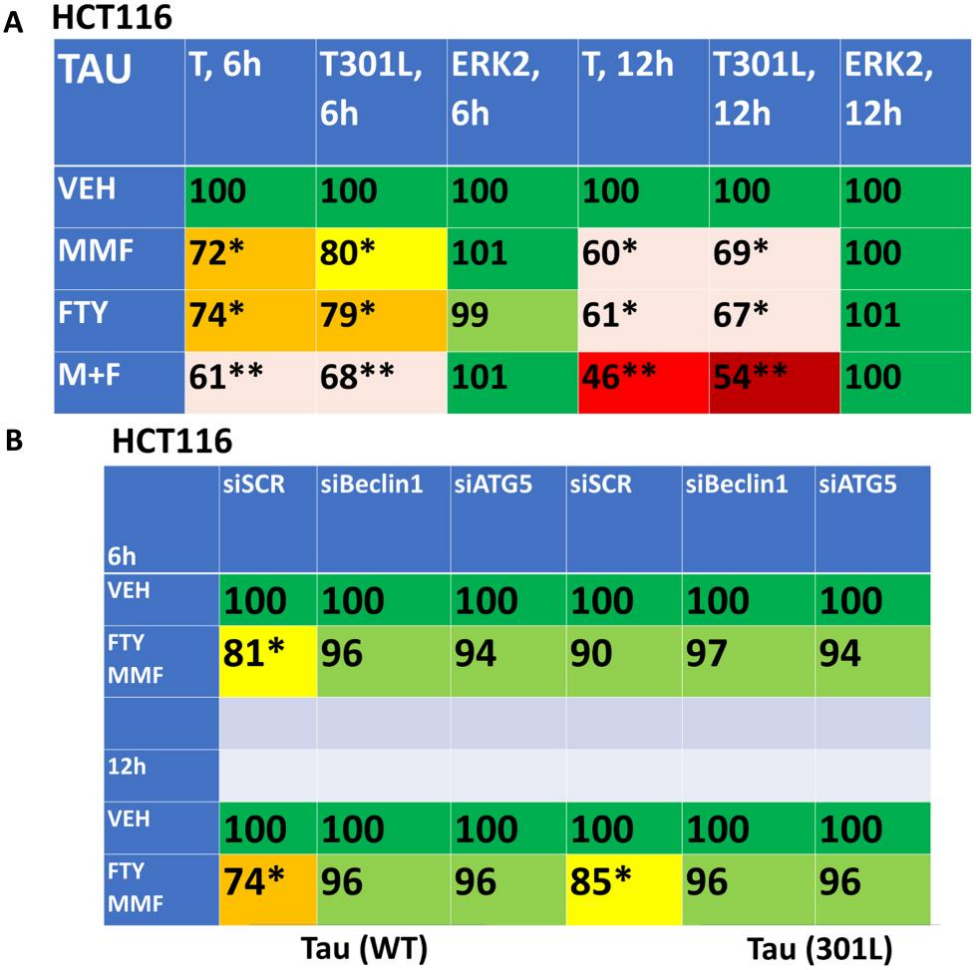
**Fingolimod and MMF interact to reduce the expression of Tau and Tau 301L via autophagy.** (**A**) HCT116 ATG16L1 T300 cells were transfected to express Tau-GFP or Tau 301L-GFP. After 24h, cells were treated with vehicle control, fingolimod (FTY, 100 nM), MMF (5 μM) or the drugs in combination for 6h or 12h. Cells were fixed in place and the expression of Tau determined by in-cell immuno-staining. (n = 3 +/-SD). * p < 0.05 less than vehicle control; ** p < 0.05 less than MMF alone value. (**B**) HCT116 ATG16L1 T300 cells were transfected to express Tau-GFP and in parallel with a scrambled siRNA control or with siRNA molecules to knock down Beclin1 or ATG5. After 24h, cells were treated for 6h or 12h with vehicle control or with [fingolimod, 100 nM plus MMF, 5 μM]. Cells were fixed in place and the expression of Tau determined by in-cell immuno-staining. (n = 3 +/-SD). * p < 0.05 less than vehicle control.

**Figure 10:**
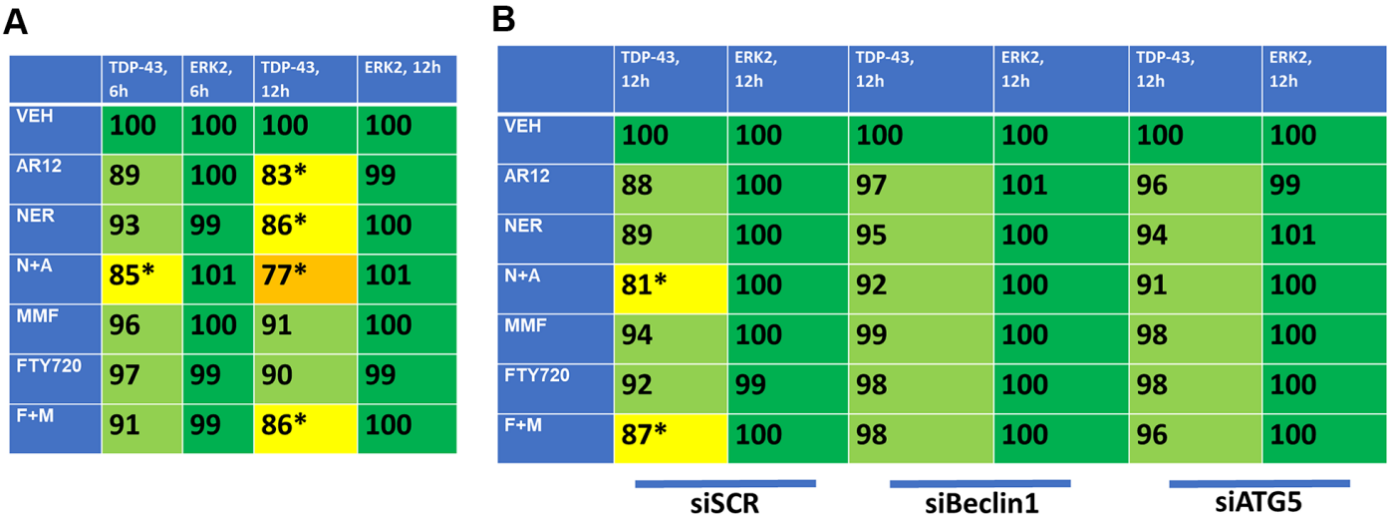
**AR12 and neratinib in combination reduce the expression of TDP-43 via autophagy.** (**A**) HCT116 ATG16L1 T300 cells were transfected to express TDP-43. Twenty-four h later, cells were treated with vehicle control, neratinib (50 nM), AR12 (2 μM), fingolimod (FTY, 100 nM), MMF (5 μM) or the drugs in combination as indicated for 6h or 12h. Cells were fixed in place and the expression of each protein plus ERK2 as a loading control determined by in-cell immuno-staining. (n = 3 +/-SD). * p < 0.05 less than vehicle control. (**B**) HCT116 ATG16L1 T300 cells were transfected to express TDP-43 and co-transfected with a scrambled siRNA or with siRNA molecules to knock down either Beclin1 or ATG5. Twenty-four h later, cells were treated with vehicle control, neratinib (50 nM), AR12 (2 μM), fingolimod (FTY, 100 nM), MMF (5 μM) or the drugs in combination as indicated for 12h. Cells were fixed in place and the expression of each protein plus total ERK2 as a loading control determined by in-cell immuno-staining. (n = 3 +/-SD). * p < 0.05 less than vehicle control.

As presented in Figure 3, fingolimod and MMF interact to cause autophagosome formation and subsequently to promote autophagic flux. As was observed for AR12 and neratinib, fingolimod and MMF interacted to reduce the expression of wild type and mutant Tau 301L proteins (Figure 9A). Knock down of Beclin1 or ATG5 prevented Tau degradation (Figure 9B). An addition protein, beyond TAU and APP, that has been linked with the biology and pathology of AD is TDP-43 [1–8]. AR12 and neratinib, and MMF and FTY720, interacted to reduce the expression of TDP-43 (Figure 10A). Knock down of Beclin1 or ATG5 prevented either combination from reducing TDP-43 levels (Figure 10B).

**Figure 11:**
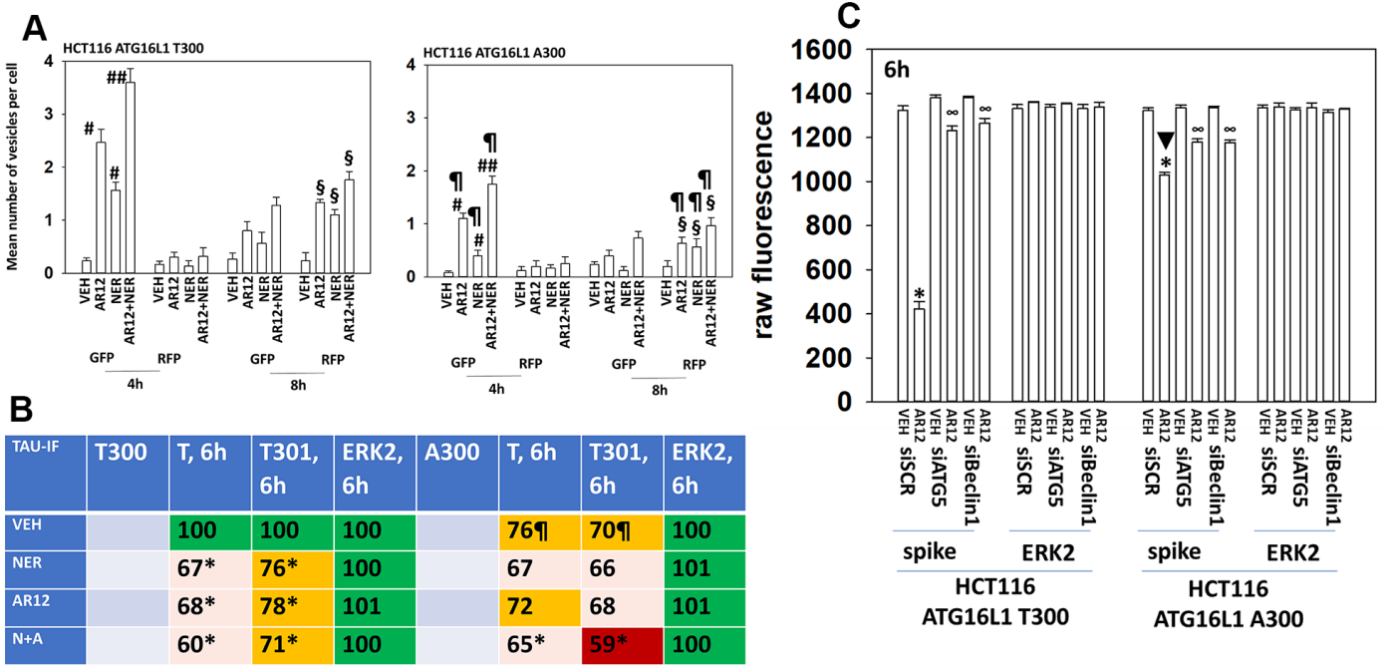
**Autophagy is compromised in HCT116 ATG16L1 A300 cells; cells that are less capable of expressing Tau.** (**A**) Isogenic HCT116 ATG16L1 T300 and HCT116 ATG16L1 A300 cells were transfected with a plasmid to express LC3-GFP-RFP. After 24h, cells were treated with vehicle control, AR12 (2 μM), neratinib (50 nM) or the drugs in combination for 4h or 8h. Randomly cells (> 50 per data point) were examined 4h and 8h after drug exposure and the mean number of GFP+ and RFP+ intense staining punctae determined under each condition (n = 3 +/-SD) # p < 0.05 greater than vehicle control; ## p < 0.05 greater than neratinib as a single agent; § greater than corresponding values at the 4h time point; ¶ p < 0.05 less than corresponding values in ATG16L1 T300 cells. (**B**) HCT116 cells (ATG16L1 T300 and ATG16L1 A300) were transfected with a plasmid to express Tau-GFP. After 24h, cells were treated with vehicle control, AR12 (2 μM), neratinib (50 nM) or the drugs in combination for 6h. Cells were fixed in place and in cell immunostaining performed to determine Tau expression (n = 3 +/-SD) * p < 0.05 less than vehicle control; ¶ p < 0.05 less than control value in ATG16L1 T300 cells. (**C**) HCT116 cells (ATG16L1 T300 and ATG16L1 A300) were transfected with a plasmid to express the SARS-CoV-2 spike protein and in parallel with a scrambled siRNA or with siRNA molecules to knock down expression of Beclin1 or ATG5. After 24h cells were treated with vehicle control or AR12 (2 μM) for 6h. Cells were fixed in place and in cell immunostaining performed to determine SARS-CoV-2 spike expression: raw fluorescence data is presented from a representative study performed in triplicate (n = 3 +/-SD) * p < 0.05 less than vehicle control; ∞ p < 0.05 greater than corresponding value in siSCR cells.

The autophagy regulatory protein ATG16L1 is expressed as two isoforms, ATG16L1 T300 and ATG16L1 A300. The ATG16L1 T300 is most commonly found in Africans and their descendants whereas the highest prevalence of ATG16L1 A300 expression is in northern Europeans and their descendants. Expression of ATG16L1 A300 predicts for a higher incidence of Crohn’s Disease in European Americans [49]. In comparison to isogenic HCT116 cells expressing ATG16L1 T300, cells expressing ATG16L1 A300 were significantly less capable of forming autophagosomes or exhibiting autophagic flux when treated with AR12 and neratinib (Figure 11A). This is similar to prior studies using other drugs / drug combinations which induce autophagosome formation. We next determined whether the differential ability of each isoform to facilitate autophagy also impacted the ability of neratinib and AR12 to cause Tau protein degradation. As would be predicted from the data in panel A, cells expressing ATG16L1 A300 were less capable of reducing Tau expression (Figure 11B). We were surprised, however, to observe also that HCT116 ATG16L1 A300 cells were less capable of expressing Tau than were their isogenic HCT116 ATG16L1 T300 counterparts. Tau is being expressed from a plasmid via a constitutive promoter. To determine whether this effect was specific to Tau, we transfected cells with a plasmid and a constitutive promoter to express the SARS-CoV-2 spike protein. The ability of ATG16L1 T300 and ATG16L1 A300 cells to express the spike protein was identical (Figure 11C). And the ability of AR12 cells to cause spike degradation was reduced in the ATG16L1 A300 cells. As African-Americans trend to present with a more rapid onset and severe Alzheimer’s disease when compared to European Americans, we postulate that ATG16L1 isoform expression may play a role.

**Figure 12:**
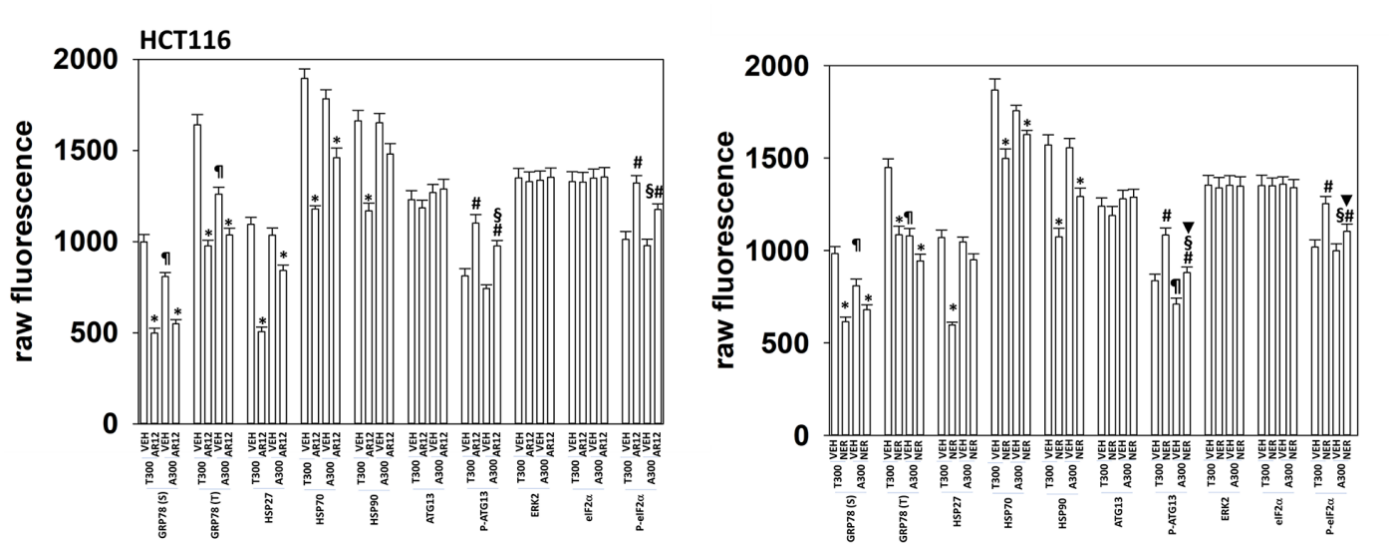
**HCT116 ATG16L1 A300 cells make less GRP78 and have a weaker stimulation of eIF2α phosphorylation by AR12 or neratinib.** HCT116 (ATG16L1 T300 or ATG16L1 A300) cells were treated with vehicle control, AR12 (2 mM) or neratinib (50 nM). Cells were fixed in place after 6h and the expression of the indicated proteins and phospho-proteins determined by in-cell immuno-staining. (n = 3 +/-SD). * p < 0.05 less than vehicle control; # p < 0.05 greater than vehicle control; ¶ p < 0.05 less than corresponding basal value in ATG16L1 T300 cells; § p < 0.05 less than corresponding stimulated value in ATG16L1 cells; ▼ p < 0.05 less than corresponding value in AR12 treated cells.

We extended our analyses of the biologies of HCT116 ATG16L1 T300 and HCT116 ATG16L1 A300 cells to determine whether altered chaperone levels, or drug responses, could provide clues as to why more Tau was expressed in the T300 cells. Regardless of isoform expression, cells expressed similar levels of HSP90 and HSP70, whereas expression of GRP78 was lower in the ATG16L1 A300 isoform cells (Figure 12). This was observed for cell surface levels of GRP78 and for bulk cellular GRP78. Although cells regardless of their isoform exhibited near identical levels of total ATG13 and total eIF2α, the phosphorylation of these proteins caused by either AR12 or neratinib was altered. In cells expressing ATG16L1 A300, the ability of AR12 and neratinib to stimulate ATG13 S318 phosphorylation (autophagy) and eIF2α S51 phosphorylation (ER stress) were reduced. Basal phosphorylation of ATG13 S318 trended towards being lower in the A300 cells (Figure 12). These findings may partially explain why cells expressing ATG16L1 A300 do not produce as many autophagosomes when stimulated as do ATG16L1 T300 cells.

**Figure 13:**
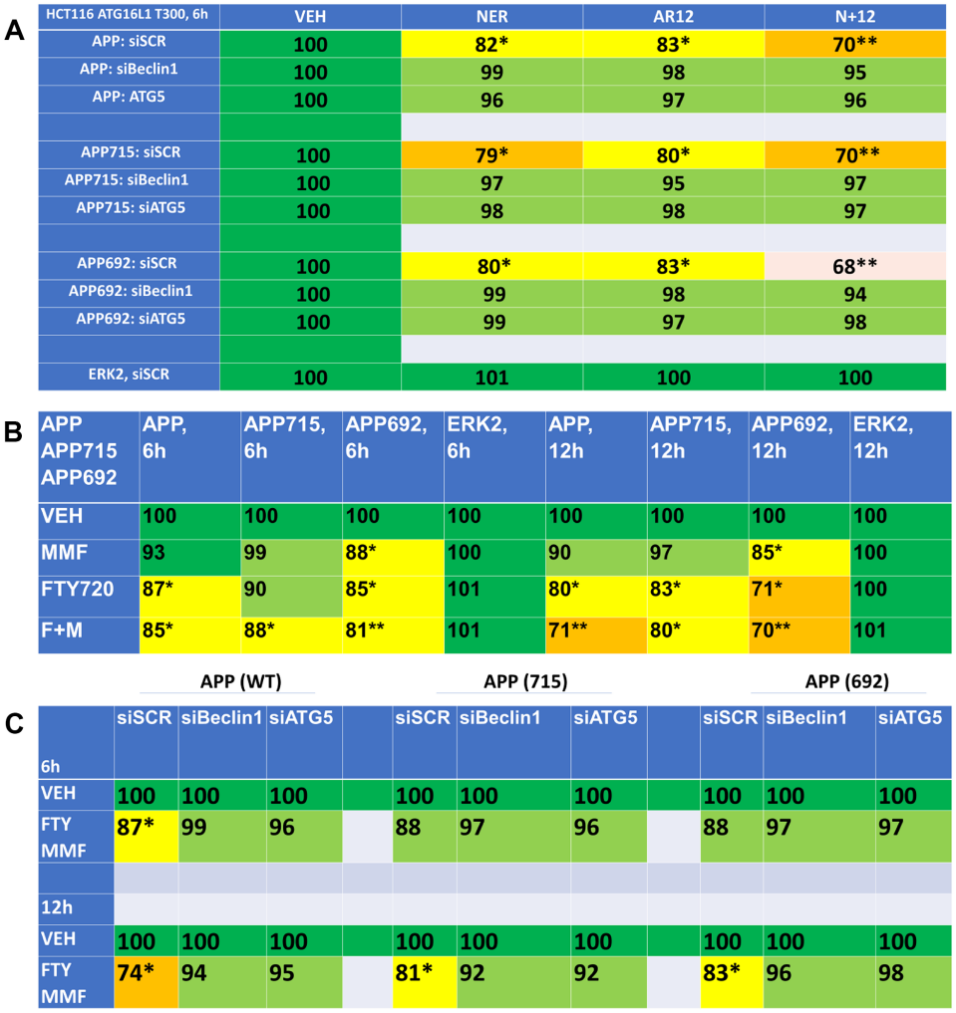
**Drug combinations reduce the expression of amyloid precursor protein (APP) via autophagy.** (**A**) HCT116 ATG16L1 T300 cells were transfected to express wild type APP or mutant APP proteins, APP715 or APP692. In parallel, cells were transfected with a scrambled siRNA control or with siRNA molecules to knock down the expression of Beclin1 or ATG5. After 24h, cells were treated with vehicle control, neratinib (50 nM), AR12 (2 μM) or the drugs in combination for 6h. Cells were fixed in place and the expression of APPs determined by in-cell immuno-staining. (n = 3 +/-SD). * p < 0.05 less than vehicle control; ** p < 0.05 less than neratinib as a single agent. (**B**) HCT116 ATG16L1 T300 cells were transfected with plasmids to express wild type APP, APP715 or APP692. After 24h, cells were treated with vehicle control, fingolimod (FTY, 100 nM), MMF (5 μM) or the drugs in combination for 6h or 12h. Cells were fixed in place and the expression of the APPs determined by in-cell immuno-staining. (n = 3 +/-SD). * p < 0.05 less than vehicle control; ** p < 0.05 less than MMF alone value. (**C**) HCT116 ATG16L1 T300 cells were transfected to express wild type APP or mutant APP proteins, APP715 or APP692. In parallel, cells were transfected with a scrambled siRNA control or with siRNA molecules to knock down the expression of Beclin1 or ATG5. After 24h, cells were treated with vehicle control or with [fingolimod, 100 nM plus MMF, 5 μM]. After 6h cells were fixed in place and the expression of the APPs determined by in-cell immuno-staining. (n = 3 +/-SD). * p < 0.05 less than vehicle control.

In addition to denaturation of Tau, AD also contains denatured beta-amyloid plaques. Hence, we next determined whether our drug combinations could reduce the expression of wild type amyloid precursor protein (APP) or of the mutant forms of this protein APP692 and APP715. AR12 and neratinib both reduced APP expression and interacted to cause a further reduction in protein levels (Figure 13A). Knock down of Beclin1 or ATG5 prevented APP degradation. As single agents, fingolimod and MMF were apparently less efficacious than AR12 and neratinib at reducing the levels of the tested APPs (Figure 13B). However, fingolimod and MMF did interact to cause a further reduction in APP expression. Knock down of Beclin1 or ATG5 also significantly reduced the ability of fingolimod and MMF to reduce APP expression (Figure 13C). Collectively, our findings in Figures 4–13 support the further evaluation of AR12, neratinib, fingolimod and MMF as repurposed treatments for pathologies such as AD.

**Figure 14:**
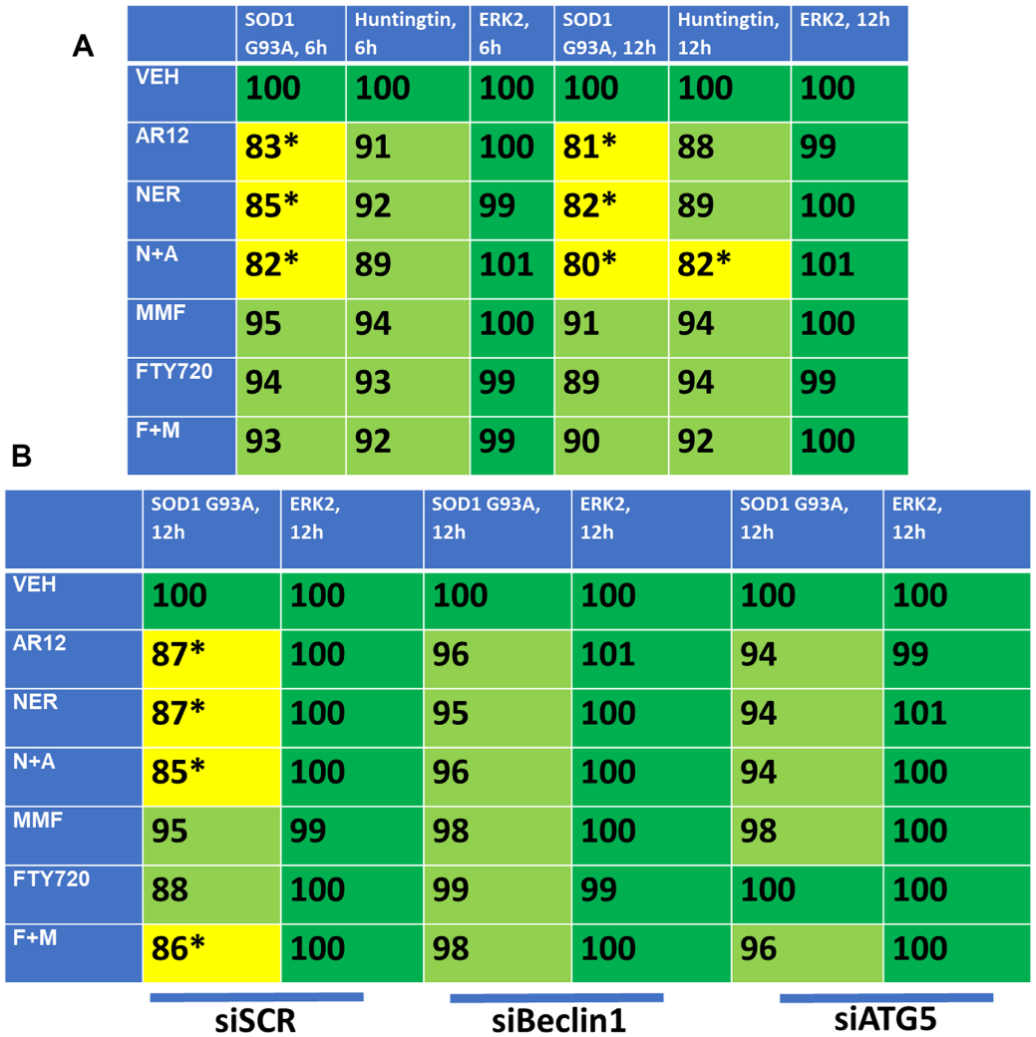
**AR12 and neratinib in combination reduce the expression of SOD1 G93A and mutant CAG repeat Huntingtin via autophagy.** (**A**) HCT116 ATG16L1 T300 cells were transfected to express SOD1 G93A or CAG 145 repeat Huntingtin. Twenty-four h later, cells were treated with vehicle control, neratinib (50 nM), AR12 (2 μM), fingolimod (FTY, 100 nM), MMF (5 μM) or the drugs in combination as indicated for 6h or 12h. Cells were fixed in place and the expression of each protein plus ERK2 as a loading control determined by in-cell immuno-staining. (n = 3 +/-SD). * p < 0.05 less than vehicle control. (**B**) HCT116 ATG16L1 T300 cells were transfected to express SOD1 G93A and co-transfected with a scrambled siRNA or with siRNA molecules to knock down either Beclin1 or ATG5. Twenty-four h later, cells were treated with vehicle control, neratinib (50 nM), AR12 (2 μM), fingolimod (FTY, 100 nM), MMF (5 μM) or the drugs in combination as indicated for 12h. Cells were fixed in place and the expression of each protein plus total ERK2 as a loading control determined by in-cell immuno-staining. (n = 3 +/-SD). * p < 0.05 less than vehicle control.

As noted previously, other lethal neurological diseases are also caused by accumulation of denatured toxic proteins including SOD1 G93A in ALS and Huntingtin in HC. Thus, based on our data with APP and Tau we determined whether our drug combinations could reduce the protein expression of SOD1 G93A and CAG 145 repeat Huntingtin. AR12 and neratinib interacted to reduce the expression of SOD1 G93A and Huntingtin, with Huntingtin being relatively less capable of being down-regulated (Figure 14A). In contrast to AR12/neratinib, and different to our findings with APP and Tau, fingolimod and MMF were ineffective at reducing protein expression. Knock down of Beclin1 or ATG5 prevented the drug-induced degradation of SOD1 G93A (Figure 14B). Additional studies will be required to understand why fingolimod and MMF did not cause significant amounts of degradation for these specific proteins.

## DISCUSSION

Toxic misfolded proteins are key drivers of AD, ALS, HC and other neurodegenerative diseases. In order to make progress against these diseases we need drugs that target these toxic proteins. In this paper we examined using isogenic colon cancer cells several existing drugs that function by increasing autophagy and degrading misfolded proteins.

Our initial studies tested AR12, neratinib, fingolimod, and MMF looking at autophagosome formation and autophagic flux. We demonstrated that AR12 and neratinib increased autophagosome formation in HCT116 cells and when these two drugs were combined showed even greater autophagosome formation. In GBM6 cells fingolimod and MMF enhanced autophagosome formation and the combination caused enhanced levels of autophagy. The increased autophagosome formation generated by these different drugs was reduced when AMPKα_1_ or eIFα were knocked down, suggesting eIF2α dependent autophagosome formation.

After confirming these drug combinations led to increased autophagosome formation we looked more specifically at their ability to degrade Tau and APP. Tau and APP levels were decreased in HCT116 cells when treated with neratinib and AR12 or fingolimod and MMF. The autophagy dependent nature of Tau and APP degradation was shown by knocking down Beclin1 or ATG5. When Beclin1 or ATG5 were knocked down using siRNA the levels of Tau and APP recovered to the same levels as the control. Similarly, in GBM6 cells we showed autophagosome degradation of Tau and APP. While many but not all researchers postulate that an aggregated form of the amyloid-beta peptide is involved in the development and potential treatment and prevention of Alzheimer’s disease and recent trial findings have raised the possibility that amyloid-beta plaque-reducing antibodies may slow clinical progression in symptomatic people, the roles of amyloid-beta and APP in the development and potential treatment and prevention if AD needs to be clarified and confirmed in additional studies.

Aberrant expression of chaperone proteins is found in many human pathologies including cancer, in virology and in AD, ALS and HC. Chaperones fall into two broad families, the HSP70 family and the HSP90 family. Thirty-years ago the chaperone HSP90 was found complexed to protein kinases such as RAF-1, where it played a key role in maintaining RAF-1 stability and its interactions with RAS proteins and MEK1/2 [55, 56]. Pharmaceutical companies have made multiple attempts to drug HSP90, however all approaches have as of the present, failed in the clinic [57, 58]. This is due to the overlapping activities of the HSP90 and HSP70 families in terms of the proteins each can chaperone. Thus, inhibition of HSP90 caused a compensatory survival response with cells increasing HSP70 levels to maintain viability. Furthermore, high efficacy drugs which bound to the ATP binding site in HSP90 did not block the ATPase activities of HSP70 family chaperones. As a therapeutic agent AR12 would have been discarded by most drug companies because it is not a low-nanomolar inhibitor of chaperone ATPases. However, AR12 has similar IC50 inhibitory efficacy against *both* HSP90 and HSP70; cells cannot compensate for chaperone inhibition as AR12 reduces the functions of both families. The relative efficacy of AR12 as a chaperone inhibitor may also explain why the drug is not toxic in non-transformed cells; because even at concentrations above 1 μM, AR12 does not completely abolish chaperone ATPase activity which very likely provides for its therapeutic window for its safe use in patients.

The chaperone whose ATPase activity is most potently inhibited by AR12 is the HSP70 family member GRP78. GRP78 plays two key roles in cell biology; it acts both as a chaperone which uses ATP to renature proteins and it acts as a sensor which assesses the levels of denatured protein in a cell. Under normal resting conditions GRP78 inhibits ER stress signaling, e.g., by blocking PERK activity and the phosphorylation of eIF2α S51. When high levels of denatured protein exist in a cell, GRP78 dissociates from PERK and associates with denatured proteins. PERK activates and phosphorylates eIF2α which prevents translation from 95% or genes and enhances translation of genes whose gene products are components of the autophagic machinery. As a more generic inhibitor of all ATP binding chaperones, AR12 also reduces mTORC1 activity, promoting autophagosome formation, flux, and the degradation of denatured proteins. As we have shown, this approach can reduce the intracellular expression of Tau and APP.

GRP78 is not only located in the cytoplasm and ER; it is also a cell surface protein, localized on the outer leaflet of the plasma membrane. For example, inhibition of plasma membrane localized GRP78 causes inactivation of the PI3K/AKT/mTOR pathway in cancer cells [59]. Plasma membrane GRP78 acts to stabilize membrane receptors, for example, acetyl choline esterase 2 (ACE2), the receptor for the SARS-CoV-2 virus [40, 60]. Our data, hence, also suggest the possibility that plasma membrane localized GRP78 may be capable of sensing the presence of denatured amyloid-β in the extracellular space liminal to the plasma membrane. Extracellular GRP78 has been shown to promote amyloid-β uptake by microglia however it is known that microglia in AD are less capable of phagocytosing denatured amyloid-β [61–65]. It has also been shown that amyloid-β induces cells to over-express GRP78 and GRP78 is over-expressed in neurons from APP/PS1 mice. The exogenous GRP78, once ingested with the amyloid-β protein, translocated to the ER of the microglia where, presumably, it would act to block ER stress signaling and the autophagic digestion of denatured amyloid-β protein. Thus, our findings raise the intriguing possibility that AR12, by blocking GRP78 function in both populations of the protein, will facilitate amyloid-β degradation in microglia as well as in neurons.

ATG16L1 is a key protein required for autophagosome formation. African-Americans trend to express the ATG16L1 T300 isoform compared to Europeans who express the ATG16L1 A300 isoform and this is causal in European Americans presenting with higher levels of Crohn’s Disease than African Americans. African Americans appear to be at greater risk of developing and presenting with a more severe form of Alzheimer’s Disease. Our initial findings were counter-intuitive to this information as we discovered that the T300 isoform is permissive for a greater ability of cells to form autophagosomes and a greater ability of immune cells to take up extracellular material, causing its degradation, thus reducing inflammation. Hence this information would argue that expression of ATG16L1 T300 would predict for microglia who will more readily ingest extracellular denatured material, e.g., amyloid-β, which would predict for less inflammation in such an Alzheimer’s brain. However, our studies also demonstrated that ATG16L1 T300 cells expressed significantly greater levels of plasma membrane associated GRP78 than did isogenic ATG16L1 A300 cells. This implies that GRP78 bound to amyloid-β will be ingested and the intracellular levels of GRP78 and denatured amyloid-β in a cell expressing ATG16L1 T300 will be relatively greater than in a cell expressing ATG16L1 A300. The levels of intracellular GRP78 will now be elevated to such an extent that this prevents ER stress signaling and autophagic degradation of the denatured amyloid-β. Furthermore, in contrast to every other protein we have previously expressed from a constitutive plasmid promoter in isogenic T300/T300 and A300/A300 cells, the protein expression of “wild type” Tau or “mutant” Tau 301L is 25% greater in T300/T300 cells compared to A300/A300 cells. These findings would a priori predict that persons homozygote for T300/T300 would present with a more severe form of AD.

The drugs tested in this manuscript have been used preclinically and clinically in several anti-cancer studies. Our present studies were performed in non-neuronal cells and as a caveat, it is possible that our data in HCT116 and Vero cells will not be reflective of the same processes in neuronal cells. As the mechanism of drug-action became clearer it was apparent that these agents should also be tested in neurodegenerative diseases. The entire neurodegenerative field needs rapid translational methods that target the underlying cause of disease, toxic misfolded protein. The findings from this work warrant further testing with a focus on clinical utility.

## References

[1] Jellinger KA. Pathobiological Subtypes of Alzheimer Disease. Dement Geriatr Cogn Disord. 2020; 49:321–33. 10.1159/000508625 33429401

[2] Masnata M, Salem S, de Rus Jacquet A, Anwer M, Cicchetti F. Targeting Tau to Treat Clinical Features of Huntington’s Disease. Front Neurol. 2020; 11:580732. 10.3389/fneur.2020.580732 33329322 PMC7710872

[3] Cheng Y, Chen Y, Shang H. Aberrations of biochemical indicators in amyotrophic lateral sclerosis: a systematic review and meta-analysis. Transl Neurodegener. 2021; 10:3. 10.1186/s40035-020-00228-9 33419478 PMC7792103

[4] Gu JL, Liu F. Tau in Alzheimer’s Disease: Pathological Alterations and an Attractive Therapeutic Target. Curr Med Sci. 2020; 40:1009–21. 10.1007/s11596-020-2282-1 33428128

[5] Portz B, Lee BL, Shorter J. FUS and TDP-43 Phases in Health and Disease. Trends Biochem Sci. 2021; 46:550–63. 10.1016/j.tibs.2020.12.005 33446423 PMC8195841

[6] Lahue RS. New developments in Huntington’s disease and other triplet repeat diseases: DNA repair turns to the dark side. Neuronal Signal. 2020; 4:NS20200010. 10.1042/NS20200010 33224521 PMC7672267

[7] Rodrigues Lima-Junior J, Sulzer D, Lindestam Arlehamn CS, Sette A. The role of immune-mediated alterations and disorders in ALS disease. Hum Immunol. 2021; 82:155–61. 10.1016/j.humimm.2021.01.017 33583639 PMC7942756

[8] Hooten KG, Beers DR, Zhao W, Appel SH. Protective and Toxic Neuroinflammation in Amyotrophic Lateral Sclerosis. Neurotherapeutics. 2015; 12:364–75. 10.1007/s13311-014-0329-3 25567201 PMC4404435

[9] Komar AA, Merrick WC. A Retrospective on eIF2A-and Not the Alpha Subunit of eIF2. Int J Mol Sci. 2020; 21:2054. 10.3390/ijms21062054 32192132 PMC7139343

[10] Humeau J, Bezu L, Kepp O, Kroemer G. EIF2α phosphorylation: a hallmark of both autophagy and immunogenic cell death. Mol Cell Oncol. 2020; 7:1776570. 10.1080/23723556.2020.1776570 32944635 PMC7469655

[11] McLaughlin M, Pedersen M, Roulstone V, Bergerhoff KF, Smith HG, Whittock H, Kyula JN, Dillon MT, Pandha HS, Vile R, Melcher AA, Harrington KJ. The PERK Inhibitor GSK2606414 Enhances Reovirus Infection in Head and Neck Squamous Cell Carcinoma via an ATF4-Dependent Mechanism. Mol Ther Oncolytics. 2020; 16:238–49. 10.1016/j.omto.2020.01.001 32128359 PMC7047134

[12] Liao Y, Gu F, Mao X, Niu Q, Wang H, Sun Y, Song C, Qiu X, Tan L, Ding C. Regulation of de novo translation of host cells by manipulation of PERK/PKR and GADD34-PP1 activity during Newcastle disease virus infection. J Gen Virol. 2016; 97:867–79. 10.1099/jgv.0.000426 26869028

[13] Elfiky AA, Baghdady AM, Ali SA, Ahmed MI. GRP78 targeting: Hitting two birds with a stone. Life Sci. 2020; 260:118317. 10.1016/j.lfs.2020.118317 32841659 PMC7442953

[14] Booth L, Roberts JL, Dent P. HSPA5/Dna K may be a useful target for human disease therapies. DNA Cell Biol. 2015; 34:153–58. 10.1089/dna.2015.2808 25689303 PMC4337461

[15] Chen W, Chan Y, Wan W, Li Y, Zhang C. Aβ_1-42_ induces cell damage via RAGE-dependent endoplasmic reticulum stress in bEnd.3 cells. Exp Cell Res. 2018; 362:83–89. 10.1016/j.yexcr.2017.11.005 29154819

[16] Cha-Molstad H, Sung KS, Hwang J, Kim KA, Yu JE, Yoo YD, Jang JM, Han DH, Molstad M, Kim JG, Lee YJ, Zakrzewska A, Kim SH, et al. Amino-terminal arginylation targets endoplasmic reticulum chaperone BiP for autophagy through p62 binding. Nat Cell Biol. 2015; 17:917–29. 10.1038/ncb3177 26075355 PMC4490096

[17] Mao K, Zhang G. The role of PARP1 in neurodegenerative diseases and aging. FEBS J. 2021. [Epub ahead of print]. 10.1111/febs.15716 33460497

[18] Migneault F, Hébert MJ. Autophagy, tissue repair, and fibrosis: a delicate balance. Matrix Biol. 2021; S0945-053X:00004–04. 10.1016/j.matbio.2021.01.003 33454422

[19] Madruga E, Maestro I, Martínez A. Mitophagy Modulation, a New Player in the Race against ALS. Int J Mol Sci. 2021; 22:740. 10.3390/ijms22020740 33450997 PMC7828440

[20] Chambraud B, Daguinot C, Guillemeau K, Genet M, Dounane O, Meduri G, Poüs C, Baulieu EE, Giustiniani J. Decrease of neuronal FKBP4/FKBP52 modulates perinuclear lysosomal positioning and MAPT/Tau behavior during MAPT/Tau-induced proteotoxic stress. Autophagy. 2021; 1. 10.1080/15548627.2021.1875611 33459145 PMC8632305

[21] Kumar S, Phaneuf D, Cordeau P Jr, Boutej H, Kriz J, Julien JP. Induction of autophagy mitigates TDP-43 pathology and translational repression of neurofilament mRNAs in mouse models of ALS/FTD. Mol Neurodegener. 2021; 16:1. 10.1186/s13024-020-00420-5 33413517 PMC7792109

[22] Wani A, Al Rihani SB, Sharma A, Weadick B, Govindarajan R, Khan SU, Sharma PR, Dogra A, Nandi U, Reddy CN, Bharate SS, Singh G, Bharate SB, et al. Crocetin promotes clearance of amyloid-β by inducing autophagy via the STK11/LKB1-mediated AMPK pathway. Autophagy. 2021; 1. 10.1080/15548627.2021.1872187 33404280 PMC8632093

[23] Zhou B, Lu Q, Liu J, Fan L, Wang Y, Wei W, Wang H, Sun G. Melatonin Increases the Sensitivity of Hepatocellular Carcinoma to Sorafenib through the PERK-ATF4-Beclin1 Pathway. Int J Biol Sci. 2019; 15:1905–20. 10.7150/ijbs.32550 31523192 PMC6743299

[24] Giovedì S, Ravanelli MM, Parisi B, Bettegazzi B, Guarnieri FC. Dysfunctional Autophagy and Endolysosomal System in Neurodegenerative Diseases: Relevance and Therapeutic Options. Front Cell Neurosci. 2020; 14:602116. 10.3389/fncel.2020.602116 33390907 PMC7773602

[25] Hegde RN, Chiki A, Petricca L, Martufi P, Arbez N, Mouchiroud L, Auwerx J, Landles C, Bates GP, Singh-Bains MK, Dragunow M, Curtis MA, Faull RL, et al. TBK1 phosphorylates mutant Huntingtin and suppresses its aggregation and toxicity in Huntington’s disease models. EMBO J. 2020; 39:e104671. 10.15252/embj.2020104671 32757223 PMC7459410

[26] Booth L, Roberts JL, Poklepovic A, Avogadri-Connors F, Cutler RE, Lalani AS, Dent P. HDAC inhibitors enhance neratinib activity and when combined enhance the actions of an anti-PD-1 immunomodulatory antibody *in vivo*. Oncotarget. 2017; 8:90262–77. 10.18632/oncotarget.21660 29163826 PMC5685747

[27] Booth L, Roberts JL, Samuel P, Avogadri-Connors F, Cutler RE, Lalani AS, Poklepovic A, Dent P. The irreversible ERBB1/2/4 inhibitor neratinib interacts with the PARP1 inhibitor niraparib to kill ovarian cancer cells. Cancer Biol Ther. 2018; 19:525–33. 10.1080/15384047.2018.1436024 29405820 PMC5927661

[28] Gordon SW, McGuire WP 3rd, Shafer DA, Sterling RK, Lee HM, Matherly SC, Roberts JD, Bose P, Tombes MB, Shrader EE, Ryan AA, Kmieciak M, Nguyen T, et al. Phase I Study of Sorafenib and Vorinostat in Advanced Hepatocellular Carcinoma. Am J Clin Oncol. 2019; 42:649–54. 10.1097/COC.0000000000000567 31305287

[29] Shafer D, Tombes MB, Shrader E, Ryan A, Bandyopadhyay D, Dent P, Malkin M. Phase I trial of dimethyl fumarate, temozolomide, and radiation therapy in glioblastoma. Neurooncol Adv. 2020; 2:vdz052. 10.1093/noajnl/vdz052 32642720 PMC7212848

[30] Yacoub A, Park MA, Hanna D, Hong Y, Mitchell C, Pandya AP, Harada H, Powis G, Chen CS, Koumenis C, Grant S, Dent P. OSU-03012 promotes caspase-independent but PERK-, cathepsin B-, BID-, and AIF-dependent killing of transformed cells. Mol Pharmacol. 2006; 70:589–603. 10.1124/mol.106.025007 16622074

[31] Booth L, Cazanave SC, Hamed HA, Yacoub A, Ogretmen B, Chen CS, Grant S, Dent P. OSU-03012 suppresses GRP78/BiP expression that causes PERK-dependent increases in tumor cell killing. Cancer Biol Ther. 2012; 13:224–36. 10.4161/cbt.13.4.18877 22354011 PMC3336069

[32] Booth L, Roberts JL, Cruickshanks N, Grant S, Poklepovic A, Dent P. Regulation of OSU-03012 toxicity by ER stress proteins and ER stress-inducing drugs. Mol Cancer Ther. 2014; 13:2384–98. 10.1158/1535-7163.MCT-14-0172 25103559 PMC4185238

[33] Booth L, Roberts JL, Cash DR, Tavallai S, Jean S, Fidanza A, Cruz-Luna T, Siembiba P, Cycon KA, Cornelissen CN, Dent P. GRP78/BiP/HSPA5/Dna K is a universal therapeutic target for human disease. J Cell Physiol. 2015; 230:1661–76. 10.1002/jcp.24919 25546329 PMC4402027

[34] Booth L, Roberts JL, Tavallai M, Nourbakhsh A, Chuckalovcak J, Carter J, Poklepovic A, Dent P. OSU-03012 and Viagra Treatment Inhibits the Activity of Multiple Chaperone Proteins and Disrupts the Blood-Brain Barrier: Implications for Anti-Cancer Therapies. J Cell Physiol. 2015; 230:1982–98. 10.1002/jcp.24977 25736380 PMC4835175

[35] Booth L, Roberts JL, Ecroyd H, Reid SP, Proniuk S, Zukiwski A, Jacob A, Damonte E, Tuñón MJ, Dent P. AR-12 Inhibits Chaperone Proteins Preventing Virus Replication and the Accumulation of Toxic Misfolded Proteins. J Clin Cell Immunol. 2016; 7:454. 10.4172/2155-9899.1000454 27957385 PMC5146995

[36] Booth L, Shuch B, Albers T, Roberts JL, Tavallai M, Proniuk S, Zukiwski A, Wang D, Chen CS, Bottaro D, Ecroyd H, Lebedyeva IO, Dent P. Multi-kinase inhibitors can associate with heat shock proteins through their NH2-termini by which they suppress chaperone function. Oncotarget. 2016; 7:12975–96. 10.18632/oncotarget.7349 26887051 PMC4914336

[37] Booth L, Roberts JL, Ecroyd H, Tritsch SR, Bavari S, Reid SP, Proniuk S, Zukiwski A, Jacob A, Sepúlveda CS, Giovannoni F, García CC, Damonte E, et al. AR-12 Inhibits Multiple Chaperones Concomitant With Stimulating Autophagosome Formation Collectively Preventing Virus Replication. J Cell Physiol. 2016; 231:2286–302. 10.1002/jcp.25431 27187154 PMC6327852

[38] Hassandarvish P, Oo A, Jokar A, Zukiwski A, Proniuk S, Abu Bakar S, Zandi K. Exploring the *in vitro* potential of celecoxib derivative AR-12 as an effective antiviral compound against four dengue virus serotypes. J Antimicrob Chemother. 2017; 72:2438–42. 10.1093/jac/dkx191 28666323

[39] Chen HH, Chen CC, Lin YS, Chang PC, Lu ZY, Lin CF, Chen CL, Chang CP. AR-12 suppresses dengue virus replication by down-regulation of PI3K/AKT and GRP78. Antiviral Res. 2017; 142:158–68. 10.1016/j.antiviral.2017.02.015 28238876

[40] Rayner JO, Roberts RA, Kim J, Poklepovic A, Roberts JL, Booth L, Dent P. AR12 (OSU-03012) suppresses GRP78 expression and inhibits SARS-CoV-2 replication. Biochem Pharmacol. 2020; 182:114227. 10.1016/j.bcp.2020.114227 32966814 PMC7502229

[41] Wang BJ, Her GM, Hu MK, Chen YW, Tung YT, Wu PY, Hsu WM, Lee H, Jin LW, Hwang SL, Chen RP, Huang CJ, Liao YF. ErbB2 regulates autophagic flux to modulate the proteostasis of APP-CTFs in Alzheimer’s disease. Proc Natl Acad Sci USA. 2017; 114:E3129–38. 10.1073/pnas.1618804114 28351972 PMC5393216

[42] Pang X, Zhao Y, Wang J, Zhou Q, Xu L, Kang D, Liu AL, Du GH. The Bioinformatic Analysis of the Dysregulated Genes and MicroRNAs in Entorhinal Cortex, Hippocampus, and Blood for Alzheimer’s Disease. Biomed Res Int. 2017; 2017:9084507. 10.1155/2017/9084507 29359159 PMC5735586

[43] Dent P, Booth L, Roberts JL, Liu J, Poklepovic A, Lalani AS, Tuveson D, Martinez J, Hancock JF. Neratinib inhibits Hippo/YAP signaling, reduces mutant K-RAS expression, and kills pancreatic and blood cancer cells. Oncogene. 2019; 38:5890–904. 10.1038/s41388-019-0849-8 31253872 PMC7133220

[44] Arrazola Sastre A, Luque Montoro M, Gálvez-Martín P, Lacerda HM, Lucia AM, Llavero F, Zugaza JL. Small GTPases of the Ras and Rho Families Switch on/off Signaling Pathways in Neurodegenerative Diseases. Int J Mol Sci. 2020; 21:6312. 10.3390/ijms21176312 32878220 PMC7504559

[45] Irwin M, Tare M, Singh A, Puli OR, Gogia N, Riccetti M, Deshpande P, Kango-Singh M, Singh A. A Positive Feedback Loop of Hippo- and c-Jun-Amino-Terminal Kinase Signaling Pathways Regulates Amyloid-Beta-Mediated Neurodegeneration. Front Cell Dev Biol. 2020; 8:117. 10.3389/fcell.2020.00117 32232042 PMC7082232

[46] Dent P, Booth L, Poklepovic A, Martinez J, Hoff DV, Hancock JF. Neratinib degrades MST4 via autophagy that reduces membrane stiffness and is essential for the inactivation of PI3K, ERK1/2, and YAP/TAZ signaling. J Cell Physiol. 2020; 235:7889–99. 10.1002/jcp.29443 31912905 PMC10324541

[47] Booth L, Cruickshanks N, Tavallai S, Roberts JL, Peery M, Poklepovic A, Dent P. Regulation of dimethyl-fumarate toxicity by proteasome inhibitors. Cancer Biol Ther. 2014; 15:1646–57. 10.4161/15384047.2014.967992 25482938 PMC4623310

[48] Dent P, Booth L, Roberts JL, Poklepovic A, Hancock JF. Fingolimod Augments Monomethylfumarate Killing of GBM Cells. Front Oncol. 2020; 10:22. 10.3389/fonc.2020.00022 32047722 PMC6997152

[49] Grimm WA, Messer JS, Murphy SF, Nero T, Lodolce JP, Weber CR, Logsdon MF, Bartulis S, Sylvester BE, Springer A, Dougherty U, Niewold TB, Kupfer SS, et al. The Thr300Ala variant in ATG16L1 is associated with improved survival in human colorectal cancer and enhanced production of type I interferon. Gut. 2016; 65:456–64. 10.1136/gutjnl-2014-308735 25645662 PMC4789828

[50] Booth L, Roberts JL, Rais R, Kirkwood J, Avogadri-Connors F, Cutler RE Jr, Lalani AS, Poklepovic A, Dent P. [Neratinib + Valproate] exposure permanently reduces ERBB1 and RAS expression in 4T1 mammary tumors and enhances M1 macrophage infiltration. Oncotarget. 2017; 9:6062–74. 10.18632/oncotarget.23681 29464055 PMC5814195

[51] Booth L, Roberts JL, Rais R, Cutler RE Jr, Diala I, Lalani AS, Hancock JF, Poklepovic A, Dent P. Neratinib augments the lethality of [regorafenib + sildenafil]. J Cell Physiol. 2019; 234:4874–87. 10.1002/jcp.27276 30203445 PMC6322207

[52] Booth L, Roberts JL, Sander C, Lalani AS, Kirkwood JM, Hancock JF, Poklepovic A, Dent P. Neratinib and entinostat combine to rapidly reduce the expression of K-RAS, N-RAS, Gα_q_ and Gα_11_ and kill uveal melanoma cells. Cancer Biol Ther. 2019; 20:700–10. 10.1080/15384047.2018.1551747 30571927 PMC6606002

[53] Zhao Z, Ho L, Suh J, Qin W, Pyo H, Pompl P, Ksiezak-Reding H, Pasinetti GM. A role of P301L tau mutant in anti-apoptotic gene expression, cell cycle and apoptosis. Mol Cell Neurosci. 2003; 24:367–79. 10.1016/s1044-7431(03)00175-1 14572459

[54] Gunawardana CG, Mehrabian M, Wang X, Mueller I, Lubambo IB, Jonkman JE, Wang H, Schmitt-Ulms G. The Human Tau Interactome: Binding to the Ribonucleoproteome, and Impaired Binding of the Proline-to-Leucine Mutant at Position 301 (P301L) to Chaperones and the Proteasome. Mol Cell Proteomics. 2015; 14:3000–14. 10.1074/mcp.M115.050724 26269332 PMC4638042

[55] Dent P, Haser W, Haystead TA, Vincent LA, Roberts TM, Sturgill TW. Activation of mitogen-activated protein kinase kinase by v-Raf in NIH 3T3 cells and *in vitro*. Science. 1992; 257:1404–07. 10.1126/science.1326789 1326789

[56] Dent P, Reardon DB, Morrison DK, Sturgill TW. Regulation of Raf-1 and Raf-1 mutants by Ras-dependent and Ras-independent mechanisms *in vitro*. Mol Cell Biol. 1995; 15:4125–35. 10.1128/MCB.15.8.4125 7623807 PMC230651

[57] Banerjee M, Hatial I, Keegan BM, Blagg BS. Assay design and development strategies for finding Hsp90 inhibitors and their role in human diseases. Pharmacol Ther. 2021; 221:107747. 10.1016/j.pharmthera.2020.107747 33245994 PMC8744950

[58] Li L, Chen NN, You QD, Xu XL. An updated patent review of anticancer Hsp90 inhibitors (2013-present). Expert Opin Ther Pat. 2021; 31:67–80. 10.1080/13543776.2021.1829595 32990109

[59] Lin YG, Shen J, Yoo E, Liu R, Yen HY, Mehta A, Rajaei A, Yang W, Mhawech-Fauceglia P, DeMayo FJ, Lydon J, Gill P, Lee AS. Targeting the glucose-regulated protein-78 abrogates Pten-null driven AKT activation and endometrioid tumorigenesis. Oncogene. 2015; 34:5418–26. 10.1038/onc.2015.4 25684138 PMC4537850

[60] Ibrahim IM, Abdelmalek DH, Elshahat ME, Elfiky AA. COVID-19 spike-host cell receptor GRP78 binding site prediction. J Infect. 2020; 80:554–62. 10.1016/j.jinf.2020.02.026 32169481 PMC7102553

[61] Kakimura J, Kitamura Y, Taniguchi T, Shimohama S, Gebicke-Haerter PJ. Bip/GRP78-induced production of cytokines and uptake of amyloid-beta(1-42) peptide in microglia. Biochem Biophys Res Commun. 2001; 281:6–10. 10.1006/bbrc.2001.4299 11178952

[62] Yuan C, Guo X, Zhou Q, Du F, Jiang W, Zhou X, Liu P, Chi T, Ji X, Gao J, Chen C, Lang H, Xu J, et al. OAB-14, a bexarotene derivative, improves Alzheimer’s disease-related pathologies and cognitive impairments by increasing β-amyloid clearance in APP/PS1 mice. Biochim Biophys Acta Mol Basis Dis. 2019; 1865:161–80. 10.1016/j.bbadis.2018.10.028 30389579

[63] Wang ZJ, Zhao F, Wang CF, Zhang XM, Xiao Y, Zhou F, Wu MN, Zhang J, Qi JS, Yang W. Xestospongin C, a Reversible IP3 Receptor Antagonist, Alleviates the Cognitive and Pathological Impairments in APP/PS1 Mice of Alzheimer’s Disease. J Alzheimers Dis. 2019; 72:1217–31. 10.3233/JAD-190796 31683484

[64] Moreno JA, Tiffany-Castiglioni E. The chaperone Grp78 in protein folding disorders of the nervous system. Neurochem Res. 2015; 40:329–35. 10.1007/s11064-014-1405-0 25107299

[65] Goswami P, Afjal MA, Akhter J, Mangla A, Khan J, Parvez S, Raisuddin S. Involvement of endoplasmic reticulum stress in amyloid β_(1-42)_-induced Alzheimer’s like neuropathological process in rat brain. Brain Res Bull. 2020; 165:108–17. 10.1016/j.brainresbull.2020.09.022 33011197

